# Infection strategy and biogeography distinguish cosmopolitan groups of marine jumbo bacteriophages

**DOI:** 10.1038/s41396-022-01214-x

**Published:** 2022-03-08

**Authors:** Alaina R. Weinheimer, Frank O. Aylward

**Affiliations:** 1 Department of Biological Sciences, Virginia Tech, Blacksburg, VA USA; 2 Center for Emerging, Zoonotic, and Arthropod-borne Pathogens, Virginia Polytechnic Institute and State University, Blacksburg, VA 24061-0913 USA

**Keywords:** Metagenomics, Microbial biooceanography, Viral genetics, Microbial ecology

## Abstract

Recent research has underscored the immense diversity and key biogeochemical roles of large DNA viruses in the ocean. Although they are important constituents of marine ecosystems, it is sometimes difficult to detect these viruses due to their large size and complex genomes. This is true for “jumbo” bacteriophages, which have genome sizes >200 kbp and large capsids reaching up to 0.45 µm in diameter. In this study, we sought to assess the genomic diversity and distribution of these bacteriophages in the ocean by generating and analyzing jumbo phage genomes from metagenomes. We recover 85 marine jumbo phages that ranged in size from 201 to 498 kilobases, and we examine their genetic similarities and biogeography together with a reference database of marine jumbo phage genomes. By analyzing Tara Oceans metagenomic data, we show that although most jumbo phages can be detected in a range of different size fractions, 17 of our bins tend to be found in those greater than 0.22 µm, potentially due to their large size. Our network-based analysis of gene-sharing patterns reveals that jumbo bacteriophages belong to five genome clusters that are typified by diverse replication strategies, genomic repertoires, and potential host ranges. Our analysis of jumbo phage distributions in the ocean reveals that depth is a major factor shaping their biogeography, with some phage genome clusters occurring preferentially in either surface or mesopelagic waters, respectively. Taken together, our findings indicate that jumbo phages are widespread community members in the ocean with complex genomic repertoires and ecological impacts that warrant further targeted investigation.

## Introduction

Although historically noted for their small virion sizes and simple genomes [1], viruses with large particles and elaborate genomes have been discovered in recent decades throughout the biosphere [2–4]. These complex viruses not only invite intriguing evolutionary questions [5–7], but also expand the potential roles viruses have in shaping microbial community structure and biogeochemical cycling [4, 8–10]. One group of these larger viruses are jumbo bacteriophages (jumbo phages), which have traditionally been defined as *Caudovirales* with genomes over 200 kilobases in length [11]. While a recent survey of cultured jumbo phages showed jumbo phages share some universal features and genes, such as encoding DNA polymerases and the terminase large subunit (TerL), these features do not distinguish them from smaller phages, and several lines of evidence suggest that jumbo phages emerged from smaller phages multiple times independently [6]. For example, a recent phylogenetic study of cultured *Caudovirales* used concatenated protein alignments to generate phylogenies and found that the most supported clades within the *Caudovirales* family do not consistently correspond to genome length [12]. Furthermore, a previous study found that jumbo phages cluster with smaller phages based on gene content and are best grouped by replication machinery, among other infection apparati [6]. Taken together, jumbo phages likely form distinct clades within the *Caudovirales*.

Although the first jumbo phages were isolated as early as the 1970s [13], these viruses have remained relatively sparse in culture, representing less than 3% (*n* = 93) of complete *Caudovirales* genomes on NCBI’s RefSeq Viral Genome Portal (downloaded July 5, 2020) and 2.2% of the INPHARED database [14]. All cultured jumbo phage capsids have morphologies of myoviruses or siphoviruses, and they undergo infection cycles that reflect temporal patterns of lytic *Caudovirales* [15, 16]. Some jumbo phages are known to stall infections resulting in "pseudolysogeny", however, which has been proposed as a competitive strategy against other phages to prevent superinfection [6]. Jumbo phages that have been studied extensively are primarily investigated for their unusually complex functional capabilities, such as encoding entire transcriptional apparati [17] or sophisticated anti-CRISPR defense mechanisms [18, 19]. Regarding their ecological range, cultivated jumbo phages have been isolated on both Gram negative and Gram positive bacteria [15], and a recent metagenomic survey uncovered these viruses in diverse, global ecosystems [4].

Despite this apparent broad environmental distribution, common methods for viral isolation and diversity surveys often bias against the inclusion of jumbo phages. Because viruses have historically been considered smaller than cells, many viral diversity surveys specifically examine only small particle sizes. For example, in plaque assays, agar concentrations are often too high for larger phage particles to diffuse through compared to smaller particles [20]. Moreover, filters are often used to remove cells when preparing viral enrichments for metagenomic sequencing [21], which excludes larger viruses [9, 22]. Particularly in marine studies, the <0.22 µm fraction, sometimes even referred to as the "viral fraction" [23], is most commonly examined for viruses [24–26]. Jumbo phages can have particles over 0.45 µm in length (i.e. *Pseudomonas aeruginosa* phage PhiKZ) [13], however, and will therefore be excluded from <0.22 µm size fractions. Lastly, in bioinformatic pipelines, phage sequences are often only assembled to the contig or scaffold level, which is sometimes sufficient for the assembly of most known smaller phage genomes [27], but often leaves larger phage genomes fragmented into multiple contigs and may require additional joining of contigs into bins [28]. Overall, considering these biases and the recently-discovered broad distribution of these viruses [4], jumbo phages may represent underappreciated components of marine microbial communities and food webs that warrant further examination.

In this study, we examine the diversity and prevalence of jumbo phages in the global ocean. We develop a workflow for generating and validating high-quality jumbo bacteriophage bins from metagenomic data with which we identify 85 bins of jumbo phages. We then compare the genetic content of these jumbo phages with other cultured phages of all sizes and metagenomic jumbo phages from other studies. We find that the jumbo phages of this study group into five distinct clusters that are distinguished by diverse replication machinery and infection strategies, implicating a broad range of potential jumbo phage-host interactions in the ocean. We then assess the distribution of jumbo phages belonging to these genome clusters in the ocean by using metagenomic data from Tara Oceans [29]. Mapping the Tara Oceans metagenomic data onto the jumbo phage sequences reveals that these jumbos phages are collectively widely distributed in the ocean, but vary in biogeography both within and between clusters, with some more enriched in surface waters relative to deeper waters and vice versa. Upon examining the collective presence of jumbo phages in different filter fractions, we also find that most could be detected in a range of different size fractions, although 34 (17 generated from this study) were recovered from only >0.22 µm fractions. Our results support the view that jumbo phages are widespread in the biosphere and may play underappreciated roles in ecosystems around the globe.

## Results and discussion

### Detection and validation of high-quality jumbo phage bins

Due to the large size of jumbo bacteriophage genomes, it is likely that they are present in multiple distinct contigs in metagenomic datasets and therefore require binning to recover high-quality metagenome-assembled genomes (MAGs) [28]. This has been shown for large DNA viruses that infect eukaryotes, where several recent studies have successfully employed binning approaches to recover viral MAGs [2, 3, 30]. Here, we used the same 1545 high-quality metagenomic assemblies [31] used in a recent study to recover giant viruses of eukaryotes [3], but we modified the bioinformatic pipeline to identify bins of jumbo bacteriophages. These metagenomes were compiled by Parks et al. [31] and included available metagenomes on the NCBI’s Short Read Archive by December 31, 2015 (see Parks et al. [31]). This dataset includes a wide variety of marine metagenomes (*n* = 469) including many non-Tara metagenomes (*n* = 165). We focused our benchmarking and distribution analyses on Tara data [29] because of the well-curated metadata and size fractions in this dataset. We first binned the contigs from these assemblies with MetaBat2 [32], which groups contigs together based on similar nucleotide composition and coverage profiles, and focused on bins of at least 200 kilobases in total length. We subsequently identified bins composed of bacteriophage contigs through analysis with VirSorter2 [33], VIBRANT [34], and CheckV [35] (see Methods for details).

The occurrence of multiple copies of highly conserved marker genes is typically used to assess the level of contamination present in metagenome-derived genomes of bacteria and archaea [36]. Because bacteriophage lack these marker genes [37], we developed alternative strategies to assess possible contamination in our jumbo phage bins. Firstly, we refined the set of bins by retaining those with no more than 5 contigs that were each at least 5 kilobases in length to reduce the possibility that spurious contigs were put together. Secondly, we assessed the possibility that two strains of smaller phages with similar nucleotide composition may be binned together by aligning the contigs in a bin to each other. Bins that had contigs with high sequence similarity across the majority of their lengths were discarded (Supplementary Fig. 1). Thirdly, we discarded bins if their contigs exhibited aberrant co-abundance profiles in different metagenomes (see Supplementary Methods). To generate these co-abundance profiles, we mapped reads from 225 marine metagenomes provided by Tara Oceans [29] onto the bins. Coverage variation between contigs was benchmarked based on read-mapping results from artificially-fragmented reference genomes present in the samples (See Methods for details). Only bins with coverage variation below our empirically-derived threshold were retained. Using this stringent filtering, we identified 85 bins belonging to jumbo bacteriophages. These bins ranged in length from 202 kbp to 498 kbp, and 31 consisted of a single contig, while 54 consisted of 2–5 contigs (Supplementary Fig. 2).

To assess global diversity patterns of jumbo bacteriophages, we combined these jumbo phage bins together with a compiled database of previously-identified jumbo phages that included all complete jumbo *Caudovirales* genomes on RefSeq (downloaded July 5th, 2020), the INPHARED database [14], a recent survey of cultivated jumbo phages [6], the Al-Shayeb et al. study [4], and marine jumbo phage contigs from metagenomic surveys of GOV 2.0 [26] (60 jumbo phages), ALOHA 2.0 [38] (8 jumbo phages), and one megaphage MAG recovered from datasets of the English Channel [39]. Ultimately, we arrived at a set of 244 jumbo phages, including the 85 bins, that were present in at least one Tara Oceans sample (min. 20% genome covered, see Methods) or deriving from a marine dataset (i.e. ALOHA, GOV 2.0) which we analyzed further in this study and refer to as marine jumbo phages. Statistics on genomic features can be found in Supplementary Dataset 1.

### Marine jumbo phages belong to distinct groups with diverse infection strategies

**Fig. 1:**
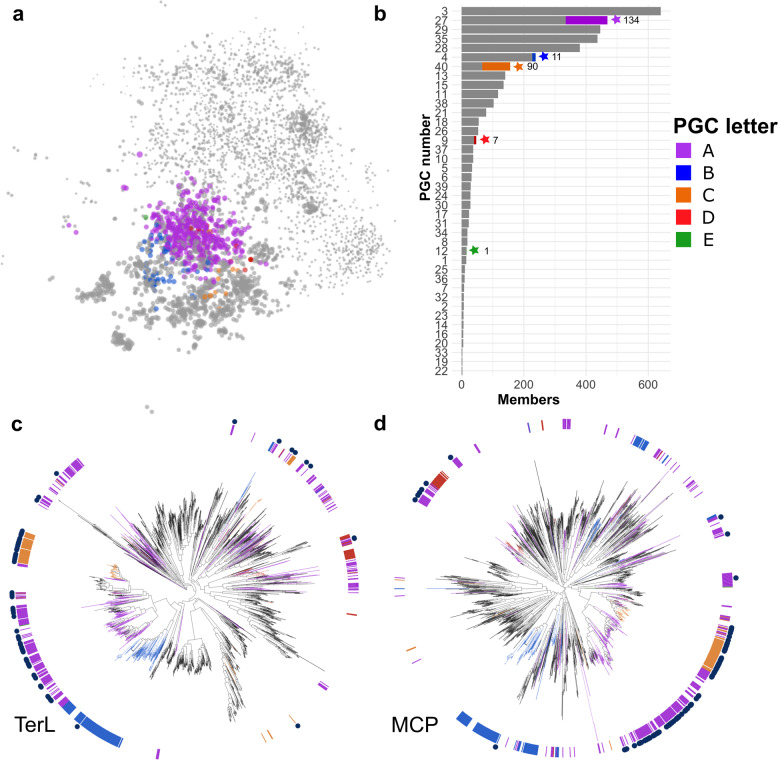
Bipartite network and marker gene analyses of jumbo phages. **a** Network with marine jumbos and references as nodes and edges based on shared VOGs. Marine jumbo phage nodes are colored by PGC as detected with spinglass community detection analysis, other nodes are in gray. Edges and VOG nodes have been omitted to more clearly represent the pattern of phage clustering. Node size corresponds to the natural log of genome length in kilobases. **b** Barchart of the number of members in each PGC. PGCs with marine jumbo phages are denoted with a star and the number of marine jumbo phages in that PGC. Proportion of marine jumbo phages in that PGC is colored. Phylogenies of TerL (**c**) and MCP (**d**) proteins with references and bins. Inner ring and branches are colored by the 5 PGCs that marine jumbo phages belong to. Navy blue circles in the outer ring denote marine jumbo phages.

**Fig. 2:**
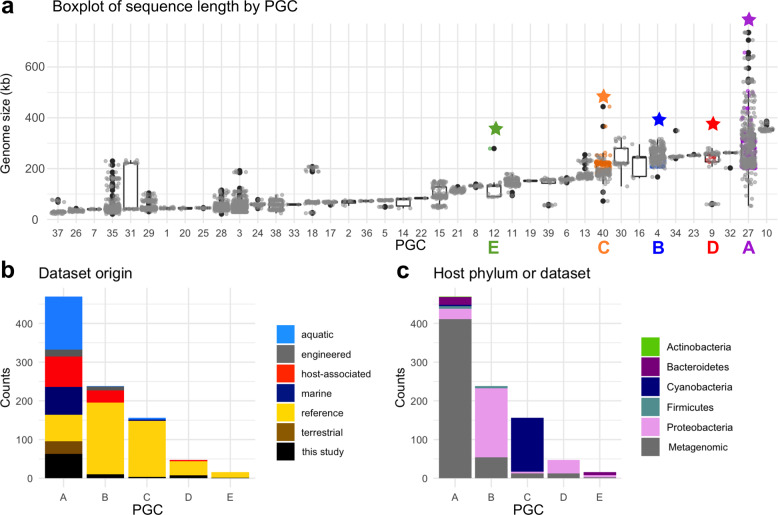
Statistics of the Phage Genomes Clusters (PGCs). **a** Boxplot of genome length in each network cluster (*x*-axis is PGC number). Star denotes PGC with a marine jumbo phage and the color matches the PGC letters of Fig. 1. **b** Stacked barplot of the metagenome environment from which each phage derives from in each PGC (*x*-axis). Reference (yellow) are cultured phages, in black are the bins of jumbo phages from this study. **c** Stacked barplot of the host phylum of the RefSeq cultured phages in each cluster; metagenomic phages are in gray.

Because bacteriophages lack high-resolution, universal marker genes for classification, such as 16S rRNA in bacteria, phages are often grouped by gene content [40, 41]. Here, we generated a bipartite network that included the 85 bins of jumbo phages with a dataset of available *Caudovirales* complete genomes in RefSeq (3012 genomes; downloaded July 5th, 2020) and the full set of reference jumbo phages described above. To construct the bipartite network, we compared proteins encoded in all the phage genomes to the VOG database, and each genome was linked to VOG hits that were present (Fig. 1, Supplementary Dataset 2, see Methods for details). To identify groups of phage genomes with similar VOG profiles, we employed a spinglass community detection algorithm [42] to generate genome clusters. Similar methods have recently been used to analyze evolutionary relationships in other dsDNA viruses [41]. The marine jumbo phages of this study clustered into five groups that included both jumbo and non-jumbo phage genomes (Fig. 2a). We refer to these five clusters as Phage Genome Clusters (PGCs): PGC_A, PGC_B, PGC_C, PGC_D, and PGC_E. These PGCs included cultured phages and metagenome-derived jumbo phages found in various environments (i.e. aquatic, engineered) and isolated on a diversity of hosts (i.e. Firmicutes, Proteobacteria, Bacteroidetes) (Fig. 2b, c). Of the marine jumbo phages, 135 belonged to PGC_A, 11 to PGC_B, 90 to PGC_C, 7 to PGC_D, and 1 to PGC_E (Fig. 1b). In addition to this network-based analysis, we also examined phylogenies of the major capsid protein (MCP) and the terminase large subunit (TerL) encoded by the marine jumbo phages and the same reference phage set examined in the network (Fig. 1c, d). With the exception of PGC_A, the marine jumbo phages that belong to the same PGC appeared more closely related to each other than those belonging to different clusters. The polyphyletic placement of jumbo phage PGCs in these marker gene phylogenies is consistent with the view that genome gigantism evolved multiple times, independently within the *Caudovirales* [6].

**Fig. 3:**
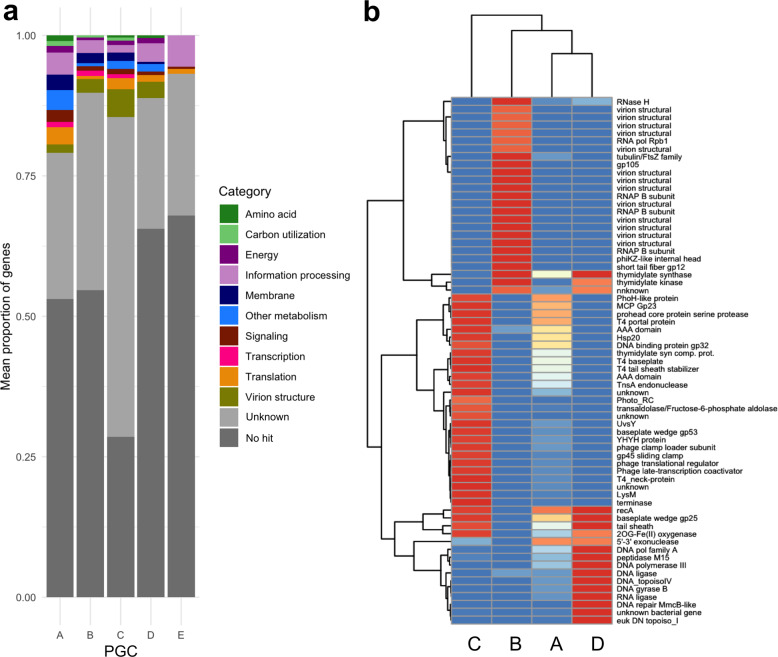
Functional predictions of PGCs. **a** Functional categories for genes encoded by jumbo phages averaged by PGC. **b** Heatmap of proportion of genomes in each PGC that contain the listed genes. Listed genes were selected based on containing a known function and having a variance between the PGCs above 0.2. Dendrogram was generated based on hierarchical clustering in pheatmap.

We then compared functional content encoded by the marine jumbo phages in the PGCs to identify functional differences that distinguish these groups. PGC_E was excluded from this analysis because this genome cluster contained only a single jumbo phage. Collectively, most genes of the marine jumbo phages could not be assigned a function (mean: 86.60%, std dev: 7.01%; Supplementary Dataset 3), which is common with environmental viruses [43, 44]. Genes with known functions primarily belonged to functional categories related to viral replication machinery, such as information processing and virion structure (Fig. 3a), and these genes drove the variation between the genome clusters of marine jumbo phages (Fig. 3b). A recent comparative genomic analysis of cultivated jumbo phages was able to identify three types of jumbo phages that are defined by different infection strategies and host interactions (referred to as Groups 1–3) [6]. We cross-referenced our PGCs and found that PGCs B, C, and D of this study corresponded to Groups 1, 2, and 3, respectively, suggesting that these genome clusters contain phages with distinct infection and replication strategies. PGC_A corresponded to multiple groups, indicating that this genome cluster contains a particularly broad diversity of phages.

The second largest phage cluster with marine jumbo phages, PGC_B, consists of 238 phages (11 (4.6%) marine jumbo phages, including 10 bins generated here), and included cultured phages of Group 1, which is typified by *Pseudomonas aeruginosa* phage PhiKZ. Supporting this correspondence with Group 1, all marine jumbo phages of PGC_B encoded the same distinct replication and transcription machinery, including a divergent family B DNA polymerase and a multi-subunit RNA polymerase (Fig. 3b, Supplementary Dataset 3). These marine jumbo phages also encoded a PhiKZ internal head protein, and they uniquely encoded shell and tubulin homologs which has recently been found in PhiKZ phages to assist in the formation of a nucleus-like compartment during infection that protects the replicating phage from host defenses [18, 19]. Although we could not confidently predict hosts for the 11 metagenomic marine jumbo phages in this PGC_B (Supplementary Dataset 1), the cultured phages of this genome cluster infect pathogenic bacteria belonging to the phyla Proteobacteria (178 phages) and Firmicutes (6 phages) (Fig. 2c), implicating a potential host range for marine jumbo phages in PGC_B.

The next largest phage genome cluster, PGC_C, comprised of 156 phages total (90 marine jumbo phages (57.7%); 4 bins generated from this study) and included reference jumbo phages in Group 2 (31, 19.9%) which are typified by Alphaproteobacteria and Cyanobacteria phages. Likewise, the host range of other cultured phages in PGC_C support the Group 2 correspondence, either infecting Cyanobacteria (139 phages) or Proteobacteria (4 phages) (Fig. 2c). Furthermore, all 3 marine metagenomic phages in PGC_C for which hosts could be predicted were matched to Cyanobacteria hosts (Supplementary Dataset 1). Functional annotations of PGC_C marine jumbo phages revealed nearly all encode a family B DNA polymerase (97.8% of phages) and the photosystem II D2 protein (PF00124, VOG04549) characteristic of cyanophages (90% of phages) (Fig. 3b). This PGC included the reference *Prochlorococcus* phage P-TIM68 (NC_028955.1), which encodes components of both photosystem I and II as a mechanism to enhance cyclic electron flow during infection [45]. This suggests that an enhanced complement of genes used to manipulate host physiology during infection may be a driver of large genome sizes in this group. Additionally, most of the PGC_C marine jumbo phages encoded lipopolysaccharide biosynthesis proteins (76%), which have been found in cyanophage genomes that may induce a "pseudolysogeny" state, when infected host cells are dormant, by changing the surface of the host cell and preventing additional phage infections [6] (Supplementary Dataset 3). Taken together, most marine jumbo phages of PGC_C likely follow host interactions of jumbo cyanophages, such as potentially manipulating host metabolism by encoding their own photosynthetic genes and potentially inducing a pseudolysogenic state.

Finally, phages of PGC_D totaled at 47 phages, of which 7 were marine jumbo phages generated in this study (14.9%). This group included Group 3 jumbo phages (15, 31.9%), which is primarily distinguished by encoding a T7-type DNA polymerase but is not typified by a particular phage type or host range. Supporting this grouping, all marine jumbo phages in this study encoded a T7 DNA polymerase which belongs to family A DNA polymerases (Fig. 3b, Supplementary Dataset 3). Most of the other genes distinctively encoded by the marine jumbo phages in this group included structural genes related to T7 (T7 baseplate, T7 capsid proteins), a eukaryotic DNA topoisomerase I catalytic core (PF01028), and DNA structural modification genes (MmcB-like DNA repair protein, DNA gyrase B). Hosts of metagenomic marine jumbo phages in PGC_D could not be predicted (Supplementary Dataset 1); however, cultured Group 3 jumbo phages in PGC_D all infect Proteobacteria, primarily Enterobacteria and other pathogens.

The largest of the phage genome clusters, PGC_A, contained 469 phages, including 135 marine jumbo phages (63 bins from this study). This genome cluster contained the largest jumbo phages, such as *Bacillus* phage G (498 kb) and the marine megaphage Mar_Mega_1 (656 kb) recently recovered from the English Channel [39]. Unlike other PGCs, PGC_A contained mostly metagenomic phages (401, 85%, Fig. 2b, c). Considering PGC_A contains the largest jumbo phages (Figs. 1b, 2a), the vast genetic diversity in this PGC might explain why few genes were found to distinguish this group. Of the genes unique to PGC_A, only one was present in at least half of the phages (51.9%), which was a Bacterial DNA polymerase III alpha NTPase domain (PF07733). The host ranges of cultured phages from this PGC further reflect the large diversity of this group and included a variety of phyla and genera that can perform complex metabolisms or lifestyles, such as the nitrogen-fixing Cyanobacteria of the *Nodularia* genus isolated from the Baltic Sea (accessions NC_048756.1 and NC_048757.1) and the Bacteroidetes bacteria *Rhodothermus* isolated from a hot spring in Iceland (NC_004735.1) [46]. Because this group contains an abundance of metagenome-derived genomes that encode mostly proteins with no VOG annotation (Supplementary Dataset 2), it is possible that it includes several distinct lineages that could not be distinguished using the community detection algorithm of the bipartite network analysis.

### Relative abundance of jumbo bacteriophages across size fractions

**Fig. 4:**
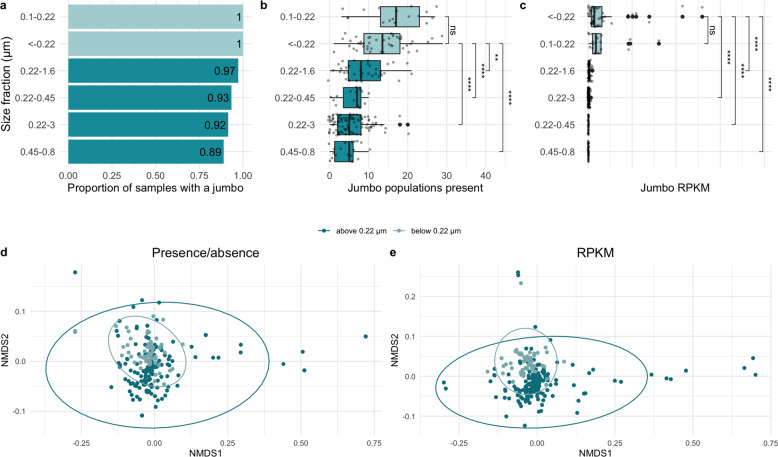
Comparison of jumbo phage abundance and presence in samples of different filter size fractions. Dark teal are fractions with minimum sizes of 0.22 μm or higher. Light teal are fractions with a maximum size of 0.22 μm or lower. **a** Barchart of the proportion of samples with at least one marine jumbo phage (*x*-axis) by size fraction (*y*-axis) sorted from highest to lowest. **b** Boxplot with *x*-axis as the number of marine jumbo phages found in a sample with size fraction on the *y*-axis sorted by median. **c** Boxplot with *x*-axis as the relative abundance of marine jumbo phages found in a sample (RPKM) with size fraction on the *y*-axis sorted by median. Significance bars in **c**, **d** correspond to Wilcox tests, with stars corresponding to *p* values < 0.05 (* < 0.05, ** < 0.01, *** < 0.001, **** < 0.0001) and those with *p* values > 0.05 as not significant "ns" (stat_compare_means function). NMDS plots (Bray Curtis dissimilarity distances) of jumbo phage composition in each sample using presence absence data (**d**) and relative abundance data (**e**). Samples are colored by size fraction distinction above 0.22 μm (dark teal) and below 0.22 μm (light teal). Ellipses calculated based on multivariate normal distribution.

To explore the distribution of the marine jumbo phages in the ocean, we first examined the size fractions in which the jumbo phages were most prevalent. To remove redundancy for the purposes of read mapping, we examined the 244 jumbo phages at the population-level (>80% genes shared with >95% average nucleotide identity [24]), corresponding to 142 populations (11 unique to this study, corresponding to 47 bins). We then mapped Tara Oceans metagenomes onto the 142 jumbo phage populations, and 102 of these populations could be detected [min. 20% of genome covered], resulting in 74 populations in PGC_A, 2 in PGC_B, 22 in PGC_C, 3 in PGC_D, and 1 in PGC_E. Out of the 225 Tara Oceans metagenomes examined, 213 (94.6%) contained at least one jumbo phage population (median: 7, Supplementary Dataset 4). Jumbo phages were more frequently detected in samples below 0.22 µm (<−0.22 µm, 0.1–0.22 µm) than those above 0.22 µm (0.45–0.8 µm, 0.22–0.45 µm, 0.22–1.6 µm, 0.22–3 µm) (Fig. 4a). All samples in the <−0.22 µm fraction and the 0.1–0.22 µm fraction had at least one jumbo phage present, while the larger fractions ranged from 89 to 97%. Interestingly, we detected 34 populations (33.3%) exclusive to samples above 0.22 µm, compared to only one population (0.98%) exclusive to samples below 0.22 µm. A similar disparity in virus detection between size fractions has been reported for large eukaryotic viruses, where roughly 41% of phylotypes were present in the 0.22–3 µm size fraction but absent in fractions below 0.22 µm [9]. In contrast to this study, where certain viral groups were more prevalent in larger size fractions than smaller, a jumbo phage’s PGC membership or genome size generally did not affect its probability of detection at different size fractions (Supplementary Figs. 3 and 4).

We also compared jumbo phage diversity (defined as population richness), relative abundance (calculated in reads per kilobase per million (RPKM)), and community composition between the size fractions (based on Bray–Curtis distance matrices). Collectively, samples of the size fractions below 0.22 µm were significantly more diverse (*p* value <0.0001, Wilcox test) and had significantly higher relative abundances (*p* value < 0.0001, Wilcox test) of jumbo phages relative to the size fractions above 0.22 µm. Despite these differences in diversity and relative abundances, jumbo phage community composition did not significantly differ between the >0.22 and <0.22 µm size fractions when comparing samples based on presence/absence data (*p* value = 0.1082, ANOSIM, presence/absence Bray–Curtis distance matrix, Fig. 4d), but did differ when using relative abundance data (*p* value = 0.0001, ANOSIM, RPKM Bray–Curtis distance matrix, Fig. 4e).

To directly test the effect of the 0.22 µm size fraction cut-off on jumbo phage recovery, we examined a subset of the samples that were co-collected at the same station and depth for the fractions below 0.22 µm (<−0.22 or 0.1–0.22) and above 0.22 µm (0.22–1.6 µm or 0.22–3 µm). The number of detected jumbo phage populations was significantly higher in samples below 0.22 µm than above 0.22 µm (*p* value = 0.000138, Wilcox test, Supplementary Fig. 5a). The relative abundance of jumbo phages was also significantly higher in size fractions below 0.22 µm than above 0.22 µm (*p* value = 0.00001, Wilcox test, Supplementary Fig. 5b). Likewise, community composition significantly differed between samples above and below 0.22 µm (*p* value = 0.0001, ANOSIM, presence/absence Bray–Curtis distance matrix, Supplementary Fig. 5c, d). Taken together, these findings suggest that using size fractions below 0.22 µm to analyze phages enhances the signal of jumbo phage sequences, relative to samples of larger size fractions, likely due to cellular sequences present in the larger sizes. Notwithstanding, roughly 33% of jumbo phages in this study were exclusive to size fractions above 0.22 µm, indicating that analyzing a range of size fractions is necessary for a more synoptic view of jumbo phages in the environment.

### Biogeography of jumbo bacteriophages in the global ocean

#### Jumbo phage populations varied by depth in different PGCs

**Fig. 5:**
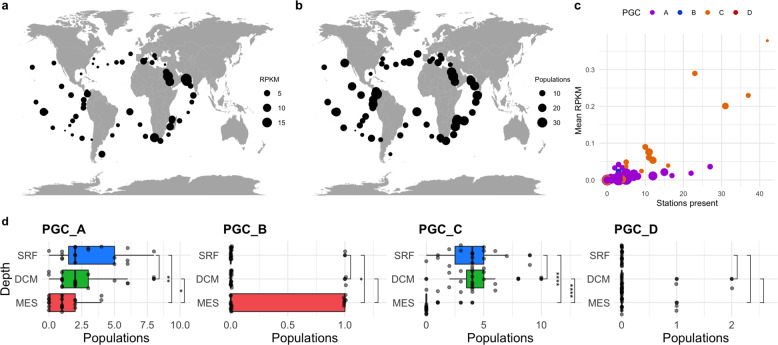
Biogeography of marine jumbo phages. Maps of the relative abundance (**a**) of total jumbo phages (in RPKM) and (**b**) total number of jumbo populations present regardless of phage cluster membership in each surface (SRF) sample of the picoplankton size fraction (either 0.22–3 μm or 0.22–1.6 μm depending on availability). Dots sizes are proportional to the number of populations or RPKM. **c** Scatterplot of the mean RPKM of a jumbo population in SRF picoplankton samples versus the number of SRF picoplankton stations it was present. Populations are colored by PGC and size corresponds to putative genome length in 100 kilobases. **d** Boxplot of the number of jumbo phage populations in samples co-collected at each depth sorted by mean for each PGC. Significance bars correspond to Wilcox tests, with stars corresponding to *p* values < 0.05 (* < 0.05, ** < 0.01, *** < 0.001, **** < 0.0001) (stat_compare_means function).

Jumbo phage populations in this study varied in their distribution (Supplementary Fig. 6) but were collectively found in all three depths (Surface (SRF), Deep Chlorophyll Maximum (DCM), Mesopelagic (MES)) examined (Fig. 5, Supplementary Fig. 7; Supplementary Dataset 4). Although jumbo phages were more prevalent in samples of the viral size fractions (<−0.22 or 0.1–0.22 µm), we focused biogeographic analyses on the 0.22–1.6 or 0.22–3 µm size fractions because the most sites were available for these samples, thereby enabling comparisons between depths and biomes. When applicable, analyses were also completed with viral fraction samples, and results are deposited at the end of the Supplement (Supplementary Figs. 16–24). Jumbo phage communities differed significantly between depths (*p* value = 0.0001, ANOSIM based on presence/absence and RPKM Bray–Curtis distance matrices, Supplementary Fig. 8), consistent with the dramatic transition in community composition that occurs from surface waters to below the deep chlorophyll maximum [38, 47]. Specifically, the diversity of jumbo phages across depths varied by genome cluster (Fig. 5d, Supplementary Fig. 10), with PGC_A and PGC_C exhibiting higher prevalence in the epipelagic (SRF and DCM). Although PGC_B and PGC_D had too few populations detected to generalize for these clusters (2 and 3, respectively), our results for these phages showed that PGC_B and PGC_D were typically less prevalent in SRF samples compared to DCM and MES samples. PGC_C is typified by cyanophages, providing a clear reason why this phage group is enriched in surface waters. Conversely, PGC_B is typified by *Pseudomonas aeruginosa* PhiKZ phages, suggesting these PGC_B marine jumbo phages may be infecting heterotrophic bacteria lineages that are less prevalent in surface waters. Overall relative abundance and diversity of jumbo phages in this study were significantly higher in the epipelagic zone (Supplementary Fig. 8), partly because most of these phages are in PGC_A. These collective and PGC-specific patterns held when examining only those samples that were co-collected at all three depths (Fig. 5c, Supplementary Figs. 8, 9, 10). This general pattern therefore reflects what has been found in previous studies on the depth distribution of viruses and viral protein clusters, where more were unique in the euphotic (i.e. epipelagic) than aphotic depths [9, 26, 48, 49], although this contrasts what has recently been found in the Pacific Ocean, where overall viral diversity increased in the mesopelagic [38].

#### Jumbo phage biogeography across biomes

Collectively, jumbo phages could be found in all three Longhurst biomes (Coastal, Westerlies, Trades) (Supplementary Fig. 11), and jumbo phage communities in this study significantly differed in composition between the biomes (*p* value < 0.05, ANOSIM, presence/absence Bray–Curtis distance matrix, Supplementary Fig. 11c). However, no biome appeared to be a particular hotspot for jumbo phages, as they were not significantly more diverse in any biome (Supplementary Fig. 11a). When looking at depths separately, the relative abundance of jumbo phages in SRF samples was significantly higher in Coastal samples (Supplementary Fig. 12b), but no clear biome was a hotspot for phages in DCM and MES samples (Supplementary Fig. 11). Likewise, jumbo phage community composition only differed between samples in the SRF and DCM, but not in MES samples (*p* value < 0.05, ANOSIM, presence/absence Bray–Curtis distance matrices). Upon examining jumbo phages by group, jumbo phages from PGCs A, C, and D could be detected in all biomes, while PGB_B phages could not be detected in Westerlies samples (Supplementary Fig. 13). Similar to their collective results, no PGC was enriched in a single biome (Supplementary Fig. 13). A recent global study on marine viruses has found that viral diversity is better explained by ecological zones defined by physicochemical factors like temperature, rather than by Longhurst biomes defined by patterns of chlorophyll *a* concentrations [26], suggesting that Longhurst biomes may not be good predictors of viral diversity in general.

#### Jumbo phage populations ranged in endemicity

Fifteen populations (14.7%) were detected in only one Tara station, and six (5.6%) were present in over half of the stations  ≥34 (Fig. 5c). Both the more endemic populations and the more prevalent jumbo phages belonged to PGCs A and C. PGC_A and PGC_C contained the most populations, which likely explains the wide range of endemicity of phages in these clusters (Supplementary Figs. 14, 15). Moreover, the cyanobacterial hosts that are known for many of the jumbo phages in PGC_C are widespread in the ocean, which may also explain the prevalence of this group of phages. In general, the heterogeneous distribution and abundance of these jumbo phages is consistent with the seed bank hypothesis, which postulates that viruses are passively dispersed throughout the ocean and viral community structure is shaped by local selective forces [24, 50]. This framework has previously been used to explain why phage distributions range from extremely cosmopolitan to extremely rare, which is a pattern that also appears to hold for jumbo bacteriophages.

## Conclusion

Large DNA viruses are becoming increasingly recognized as critical components of the virosphere, notable for their intriguing evolutionary histories [51, 52], vast functional capacities [3, 4], and global distribution [2, 4]. Here, we assess the diversity and ecology of marine jumbo bacteriophages, which have historically been difficult to study due to biases in filtration and isolation strategies. We employed a binning strategy to generate and quality-check genomes of jumbo phages and used it to identify 85 high-quality bins. We employed a conservative approach to genome binning because binning has traditionally not been used for bacteriophages, and as a result these bins likely represent a small fraction of total jumbo phages in these marine samples. We combined these bins together with reference jumbo bacteriophage genomes, and ultimately identified 102 populations that are present in Tara Oceans metagenomes. When compared with other metagenomic jumbo phages and cultured phages of all sizes, we found that marine jumbo phages primarily belong to four phage genome clusters (PGCs) that largely encode distinct replication machinery, biogeography, and potential hosts. For example, marine jumbo phages in PGC_C follow cyanophage infection strategies and ecology, as this cluster included cultured marine cyanophages and encoded classic family B DNA polymerases and photosynthesis enzymes characteristic of cyanophages. Furthermore, we found they are enriched in surface waters relative to the mesopelagic, consistent with the geographic range of their hosts. In contrast, marine jumbo phages of PGC_B included cultured PhiKZ phages of *Pseudomonas aeruginosa* and uniquely encoded multi-subunit RNA polymerases and tubulin, which are thought to play a role in the remarkable nucleus-like structures that these viruses employ as an anti-CRIPSR defense [18, 19]. PGC_B was more often found in mesopelagic waters, suggesting that this complex infection strategy may be more common in the deep ocean. PGC_A contained a large number of metagenome-derived viruses and was not as well-defined as the other clusters; it is possible that this cluster contains several distinct lineages, and more in-depth analyses will be required to assess. Overall, these results suggest that jumbo phages exhibit diverse biology and ecology, consistent with the view that they are an incredibly diverse set of phages with unique evolutionary histories [6].

The jumbo phages we analyze are collectively widespread throughout the ocean and are typically more diverse and abundant in epipelagic waters, which reflect previous findings that surface waters usually harbor a higher per-sample alpha diversity of viral groups compared to deeper waters [9, 26]. Larger phages therefore appear to coexist in patterns broadly similar to smaller viruses despite the disadvantages of their size, such as smaller burst sizes and lower host contact rates [53]. In eukaryotic giant viruses, it has been hypothesized that these disadvantages are potentially offset by higher infection efficiency, broader host ranges, decreased decay rates, and higher rates of successful attachments compared to smaller viruses [53]. Although some of these advantages to viral gigantism may also apply to jumbo bacteriophages, it is unlikely that they are all applicable. For example, given that they are tailed *Caudovirales* [6], jumbo phages likely possess higher host specificity in part due to their non-phagocytotic mode of infection. Nonetheless, the large genomes of jumbo bacteriophage often encode an expanded complement of genes used to manipulate host physiology during infection, and these may play critical roles in promoting infection efficiency or offsetting host defense mechanisms. The impressive complement of photosynthesis genes in PGC_C is at least partially responsible for the large genomes in this lineage, while the genes involved in anti-CRISPR defense found in PGC_B indicate that a host-virus arms race may be responsible for genome gigantism in this group. Interestingly, the largest number of jumbo phage genomes we identified belong to PGC_A, which is largely uncharacterized and composed of primarily metagenome-derived genomes, suggesting that these viruses have as-yet unidentified infection strategies. Overall, it is likely that the factors leading to and maintaining genome gigantism in each of these genome clusters are distinct. Future work further characterizing the hosts of these jumbo phages and the details of their infection programs, particularly in PGC_A, will therefore be critical to understanding mechanisms that underlie complexity in the virosphere and maintain diversity. Moreover, future in-depth examination of the genomics and evolutionary histories of jumbo phages will be an important step to integrating these viruses into a meaningful taxonomy and clarifying their evolutionary relationships to other *Caudovirales*.

### Methods summary

#### Jumbo phage binning and detection

An overview of the pipeline can be found in Supplementary Fig. 25. Metagenomic scaffolds were downloaded from 1545 assemblies by Parks et al. [31] and binned with MetaBAT2 [32] (-s 200000 -unbinned -t 32 -m 5000 -minS 75 -maxEdges 75) using the coverage files provided by Parks et al. Bins were retained if they summed to at least 200,000 base-pairs and comprised < = 5 contigs (min. contig size 5 kb). Proteins were predicted with Prodigal [54] using default settings on each bin individually. Bins were retained if they lacked more than one ribosomal protein, lacked overlapping regions (via promer and gnuplot [55] with MUMmer 3.0 [56]), had fewer hits to NCLDV than phage (via LASTp [57] against RefSeq r99), lacked more than one NCLDV marker gene (via hmmsearch (hmmer.org) against NCLDV marker gene HMM profiles [58]) and had even read coverage of Tara Ocean metagenomes via coverM [59] (https://github.com/wwood/CoverM). Briefly, "even read coverage" means that the read coverage of each contig in the bin varied between one another below a variation threshold determined by reference mapping results (See Supplementary Methods for details). Jumbo phages were then detected by running the bins through VirSorter2 [33], VIBRANT [34], and CheckV [35]. Bins were considered putative phages if they had at least an average dsDNAphage score of >0.9 from VirSorter2 or a VirSorter2 average score >0.5 and either (i) CheckV quality of medium or higher or (ii) VIBRANT consensus classification as viral. Ultimately, 85 bins were retained for downstream analyses. Prior to further gene-based analyses, we checked if the jumbo bins used alternative genetic codes with Codetta [60], and all were found to use the standard bacteria code 11; we therefore proceeded with the initial Prodigal predictions. Bins were grouped into populations based on single-linkages with a compiled set of 898 jumbo bacteriophages (RefSeq phages over 200 kilobases (93), phage sequences over 200 kilobases from the INPHARED database (354) [14], Iyer et al 2021 (46, non-overlapping with INPHARED) [6], Al-Shayeb et al. 2020 [4] jumbo phage genomes (336), GOV 2.0 (60) [26], ALOHA 2.0 (8) [38], and a megaphage assembled from the English Channel [39]) based on nucleotide sequences of genes (predicted with prodigal -d flag) aligned with BLASTn [61] (>95% average nucleotide identity, >80% genes) [24]. See Supplementary Methods for details.

#### Bipartite network and phylogenetic analyses

Because phages lack universal, high-resolution phylogenetic marker genes, gene-sharing networks have typically been used to classify phages [41, 62]. Bipartite networks are commonly used to examine evolutionary relationships in divergent viral lineages [3, 41]. To classify the jumbo bins of this study with a bipartite network, reference phage sequences were compiled from RefSeq’s *Caudovirales* complete genomes (downloaded July 2020 from NCBI’s Virus genome portal; 3012 genomes) along with the curated jumbo phage set used in the population analysis. Proteins of jumbo bins and this reference set were predicted with Prodigal and searched against the Virus Orthologous Groups (VOGs, vogdb.org) via HMM searches (*E* value 0.001). A bipartite network was made based on shared VOGs using igraph (graph.incidence) (1.2.5) [63] in R (version 3.5.1) [64] with RStudio (version 1.1.456) [65]. A previous study classified divergent viral lineages via the spinglass community detection algorithm [42], which we used on the bipartite network generated here via igraph (50 spins for 100 iterations). Final clusters were determined by using those of the iteration with the highest modularity. Plots of the cluster composition from the bipartite network analysis were made with ggplot2 (3.1.1) [66] in R with Rstudio. TerL (terminase large subunit) and MCP (major capsid protein) trees were made with hits to TerL VOG families and MCP VOG families encoded by the jumbo phages and reference hits (hmmsearch, *E* value < 0.001; See Data Availability). Reference hits were de-replicated with CD-HIT (version 4.8.1 -c 0.9) [67] and filtered for size (See Supplementary Methods). Proteins were then aligned with Clustal Omega [68], trimmed with trimAl (-gt 0.1) [69] and constructed with IQ-TREE [70] (TEST model selection with ModelFinder [71]).

#### Size fraction and ecological analyses

Metagenomic reads from Tara Oceans were trimmed with trim_galore (-paired -length 50 -e 0.1 -q 50) and subsampled to an even depth of 20 million reads per sample with seqkit sample (-s 1000 -n 20000000 -2). These reads were then mapped onto the population representatives of the jumbo phage set (535 populations). For the mapping, the reference database of the representative jumbo phage sequences was created with minimap2 (minimap2 -x sr -d) [72], and the mapping was carried out with coverM (coverm genome -min-read-percent-identity 95 -m covered_fraction rpkm count variance length -t 32 -minimap2-reference-is-index -coupled)). Mapping results were retained if at least 20% of the phage genome was covered (see Supplementary Methods for benchmarking, Supplementary Fig. 26). Relative abundance of a phage in each sample was calculated in RPKM. Statistical analyses and plots were carried out in R with vegan (2.5-5) [73], ggplot2, maps (3.3.0) [74], and ggpubr (0.2.4) [75] packages. Community composition was compared between variables using ANOSIMs based on Bray–Curtis distances using both presence/absence and RPKM matrices with a significance *p* values < 0.05. Statistical tests were carried out with the ggplot2 function stat_compare_means(label="p.signif").

#### Annotation

Amino acid sequences of genes were annotated with HMM searches (*E* value < 0.001) against the Pfam [76] (version 32), eggNOG (5.0) [77], and VOG (release 98) databases. Virion structural protein families were identified based on VOG hit descriptions (Supplementary Dataset 3). Consensus annotation was based on Pfam annotations and then the highest bit score between eggNOG and VOG hits. Functions were grouped into larger categories (Supplementary Dataset 3). Clusters from the network analyses were compared for functional composition between marine jumbo phages by averaging the proportion of genes in a functional category (Fig. 3a). Genes with the highest variance between PGCs were identified based on the variance in the proportion of genomes in a PGC with that gene. Those with a variance >0.2 and a known function were visualized with pheatmap [78] in R (Fig. 3b).

#### Host prediction

Hosts of the jumbo phage bins were predicted based on matching CRISPR spacers, tRNAs, and gene content. CRISPR spacers were predicted on the Genome Taxonomy Database (release 95) [79], MAGs of bacteria and archaea from the metagenomes that the jumbo phage bins derived (provided by Parks et al. [31]), and on the jumbo phage bins with minCED (derived from reference [80]). Spacers were aligned with BLASTn (-task blastn-short) and matches were >24 bp with <= 1 mismatches [4]. Only one jumbo phage contained a CRISPR array, but the spacers did not match any other jumbo phages or MAGs. tRNA sequences were predicted with tRNAscan-SE (-bacteria option) [81] on the MAGs and jumbo phage bins. Promiscuous tRNAs [82] were removed (BLASTn hits 100% ID, <= 1 mismatches). Jumbo phage tRNAs were aligned against the MAGs tRNAs and NCBI nr database (BLASTn 100% ID, <= 1 mismatches) [4]. Lastly, hosts were assigned based on the taxonomy of coding sequence matches to the MAGs (BLASTn). Hits to phyla were summed and a putative host phylum had three times the number of hits as the phylum with the next most hits as used in a previous study [4]. If a putative host could be predicted by multiple methods, a consensus host was assigned if all approaches agreed on a phylum. If the methods disagreed at the phylum-level, no putative host was assigned.

## Supplementary information

Supplemental Material

Supplemental Dataset 1

Supplemental Dataset 2

Supplemental Dataset 3

Supplemental Dataset 4

## Data Availability

Nucleic acid sequences and protein predictions for the 85 bins analyzed in this study and the proteins and files for the phylogenetic analyses (proteins, HMM profiles, treefiles) can be found on FigShare (https://figshare.com/projects/Marine_jumbo_phages/127391).
