# Supplementary material for: Infection strategy and biogeography distinguish cosmopolitan groups of marine jumbo bacteriophages
Source: ISME J. 2022 Mar 8;16(6):1657–67. doi: 10.1038/s41396-022-01214-x (PMC9123017; doi:10.1038/s41396-022-01214-x)
Supplement: Supplementary file 1 — Supplemental Material [file 41396_2022_1214_MOESM1_ESM.pdf]

Supplemental Information

Supplemental Dataset 1: Excel file of marine jumbo phage genomic features (length, contigs, population representatives), host predictions, network cluster membership, Iyer group designation, jumbo phage detection results.

Supplemental Dataset 2: VOG matrix used to generate network and cluster membership of all sequences in network, table features of phage sequences (i.e. cluster membership) used to annotate network.

Supplemental Dataset 3: Protein annotation file; sheet 1 contains marine jumbo phage protein hits to EggNOG, VOG,and Pfam; sheet 2 contains category descriptions; sheet 3 specifies virion structure VOGs.

Supplemental Dataset 4: Read mapping results (counts, fraction covered, RPKM, presence/absence); sample metadata (i.e. longitude, latitude, biome); list of genomes used for benchmarking genome coverage threshold.

Supplemental Figures

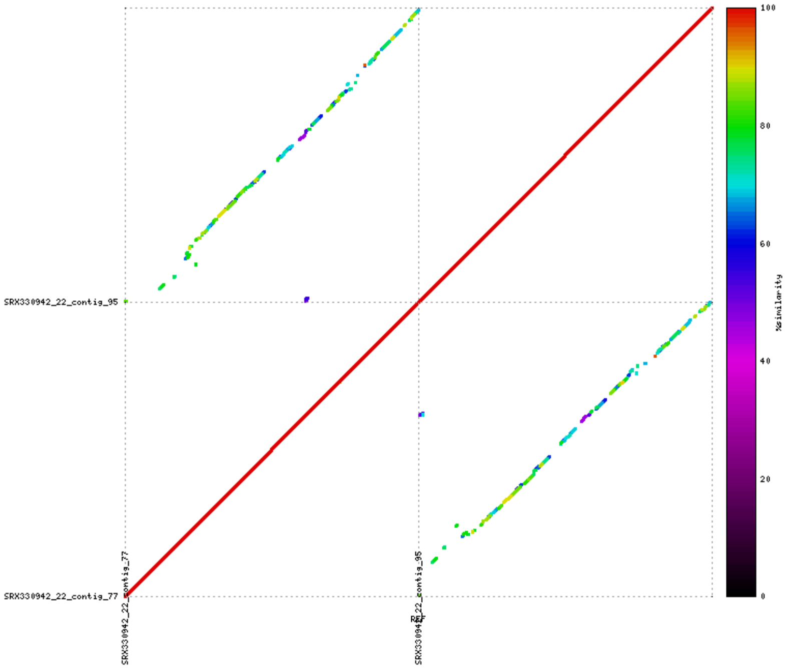

Supplemental Figure 1. Example mummerplot of promer alignment between contigs of a single bin. The top right quadrant shows the alignment of the top contig to the bottom contig and the top left quadrant shows the alignment of the top contig to itself. The color of the line corresponds to percent identity. Diagonal lines in the top left and bottom right quadrant show the

two contigs of this bin align to each other across their entire lengths with relatively high percent identity, suggesting these contigs belong to two smaller phages, rather than a single jumbo phage genome.

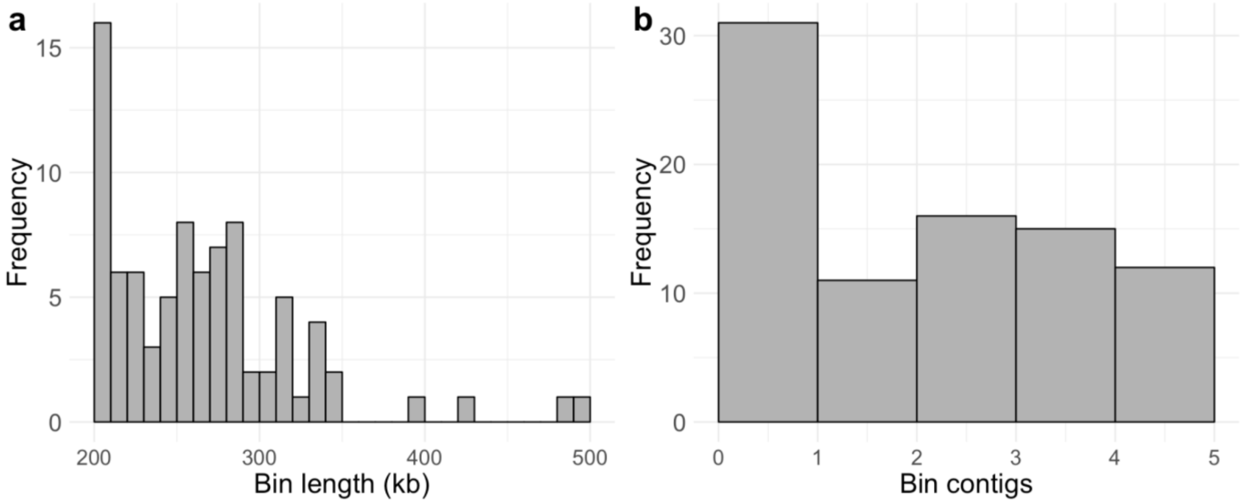

Supplemental Figure 2. (a) Histogram of jumbo bin lengths **(b)** Histogram of the number of contigs in each bin

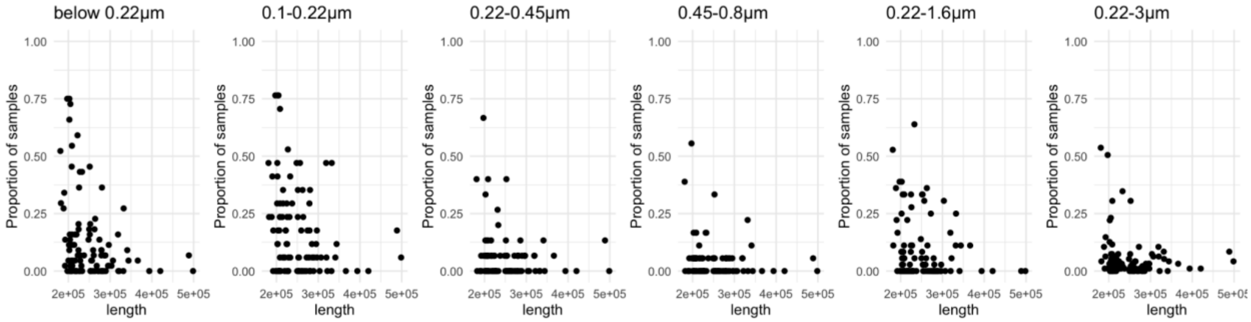

Supplemental Figure 3. Scatterplot with proportion of samples at different size fractions that a jumbo phage is present (y-axis) vs. its length (x-axis)

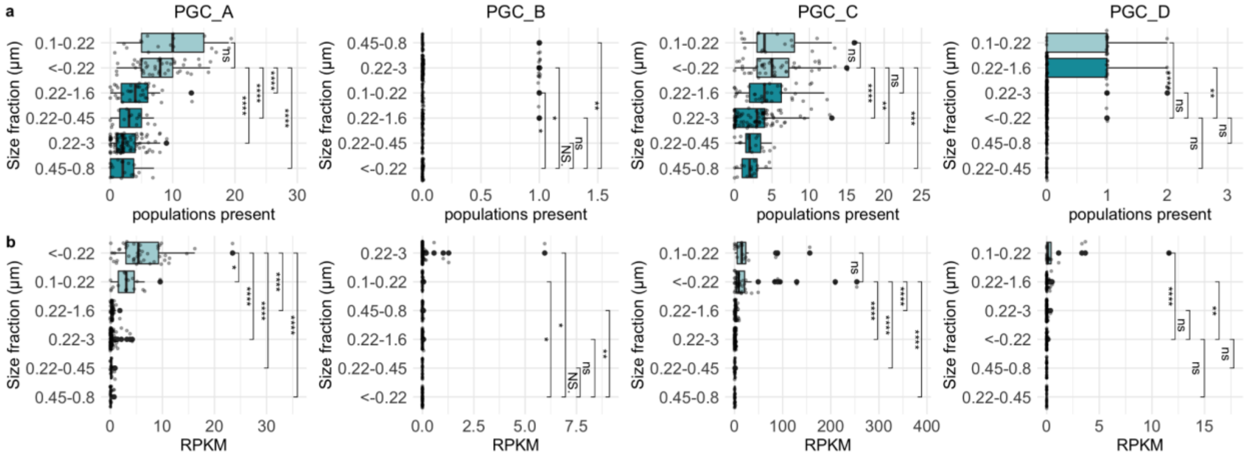

Supplemental Figure 4. **(a)** boxplots for each PGC of the number of jumbo phage populations in each sample of different size fractions sorted by mean.**(b)** boxplots for each PGC of the relative abundance of jumbo phages (RPKM) in each sample of different size fractions sorted by median. Significance bars correspond to Wilcox test, with stars corresponding to *p* values < 0.05 and those with *p* values > 0.05 as not significant "ns" (ggplot2 stat_compare_means function).

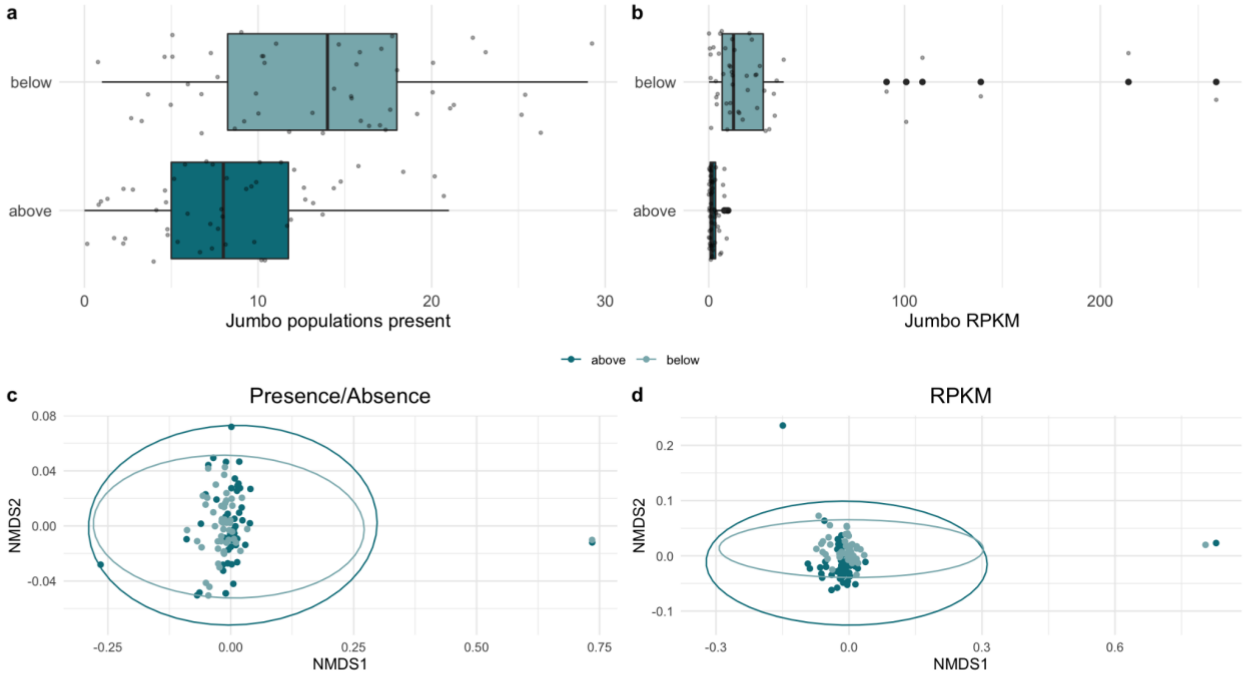

Supplemental Figure 5. (**a**) Boxplot of the number of jumbo phage populations present co-collected at the same station and depth but filtered at below 0.22 µm (size fractions "<-0.22µm" or "0.1-0.22µm") and above 0.22 µm (size fraction "0.22-1.6µm" or "0.22-3µm") (**b**) boxplot of the total RPKM of jumbo phages in these samples. (**c**) NMDS plot of samples based on Bray-Curtis distance matrices of jumbo populations' presence/absence (Richness); communities significantly differed between above and below 0.22 (*p* value = 0.0001, ANOSIM Statistic R

0.1178) (**d**) NMDS plot based on jumbo populations' RPKM; communities significantly differed between above and below 0.22 (*p* value = 0.0001, ANOSIM Statistic R 0.2229). Ellipses calculated based on multivariate normal distribution.

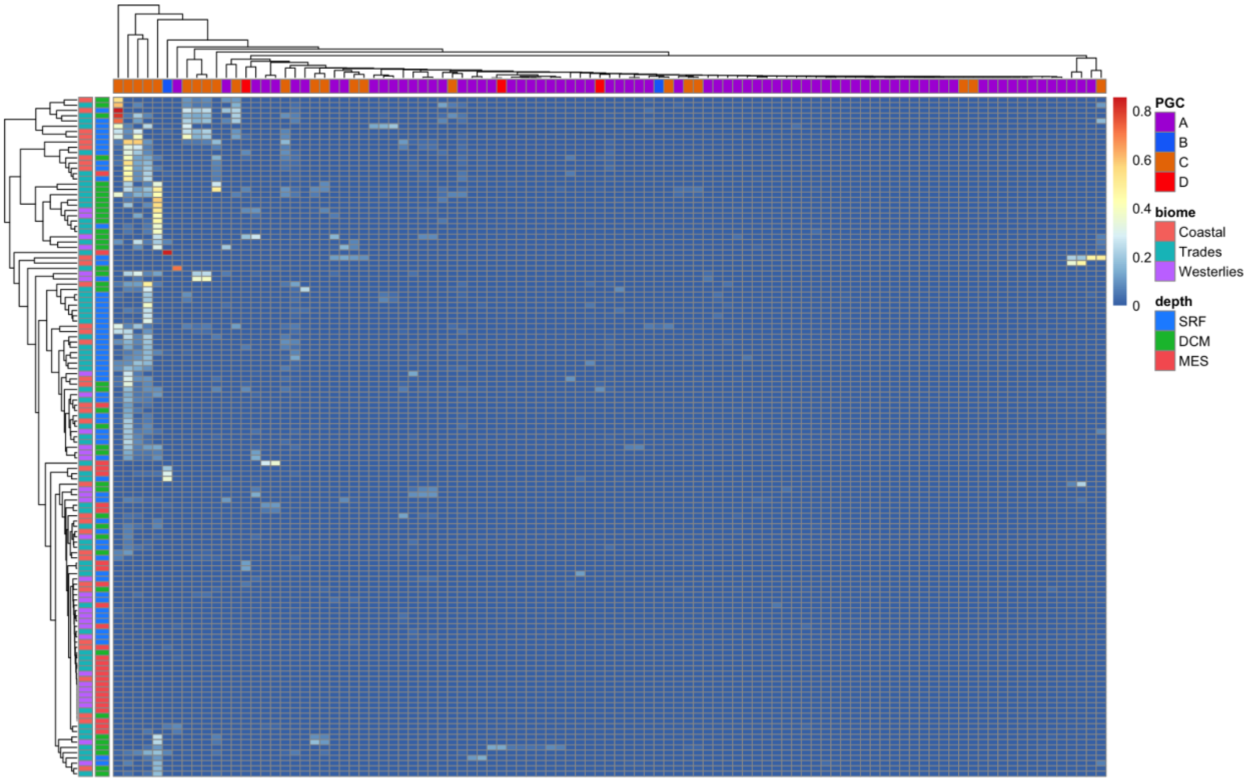

Supplemental Figure 6. Heat map of the log-transformed RPKM (log10(1+RPKM)) of each jumbo phage from this study (columns) in each picoplankton sample (rows). Rows and columns are clustered using hierarchical clustering via pheatmap default settings. Row annotation strip corresponds to each phage's PGC. The outer annotation strip on the columns corresponds to a sample's biome and the inner strip corresponds to a sample's depth.

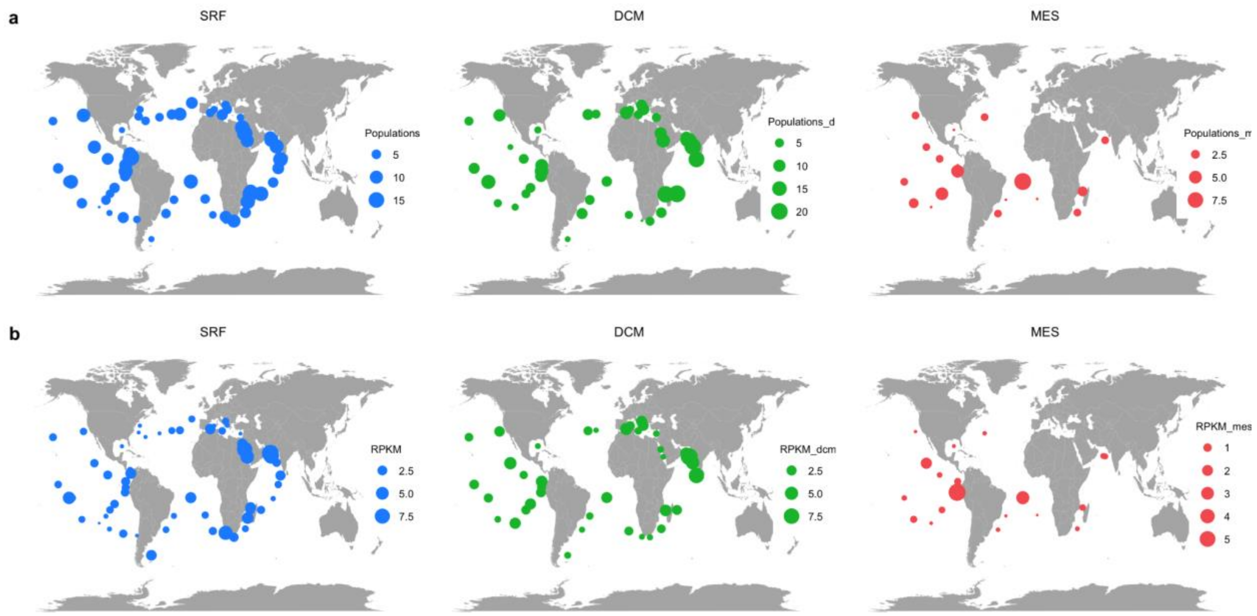

Supplemental Figure 7. Distribution of picoplankton fraction (0.22-1.6 μm or 0.22-3 μm) at each depth. Points are colored by depth (SRF - blue, DCM - green, MES - red). Point sizes in upper row maps correspond to the number of jumbo populations in a sample and point sizes in bottom row maps correspond to jumbo relative abundance (RPKM).

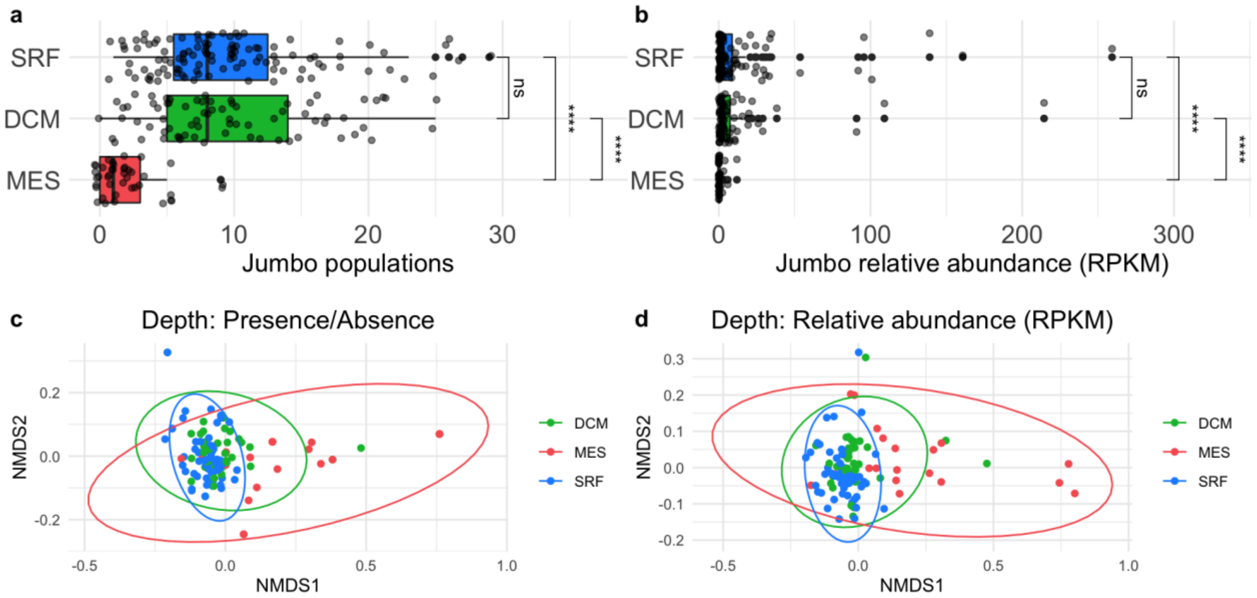

Supplemental Figure 8. (a,b) boxplots of jumbo populations present **(a)** and jumbo abundance in RPKM **(b)** in picoplankton samples by depth sorted by median abundance. Significance bars for a,b correspond to Wilcox test, with stars corresponding to p values < 0.05 and those with p values > 0.05 as not significant "ns" (ggplot2 stat_compare_means function). **(c,d)** NMDS plots of jumbo composition in those samples based on Bray-Curtis dissimilarity distances using jumbo populations' presence/absence data **(c)** and jumbo population relative abundance in RPKM **(d)**

colored by depth. Green - DCM, red - MES, blue - SRF. Ellipses calculated by multivariate normal distribution. Depths were significantly different using ANOSIM (p values < 0.01).

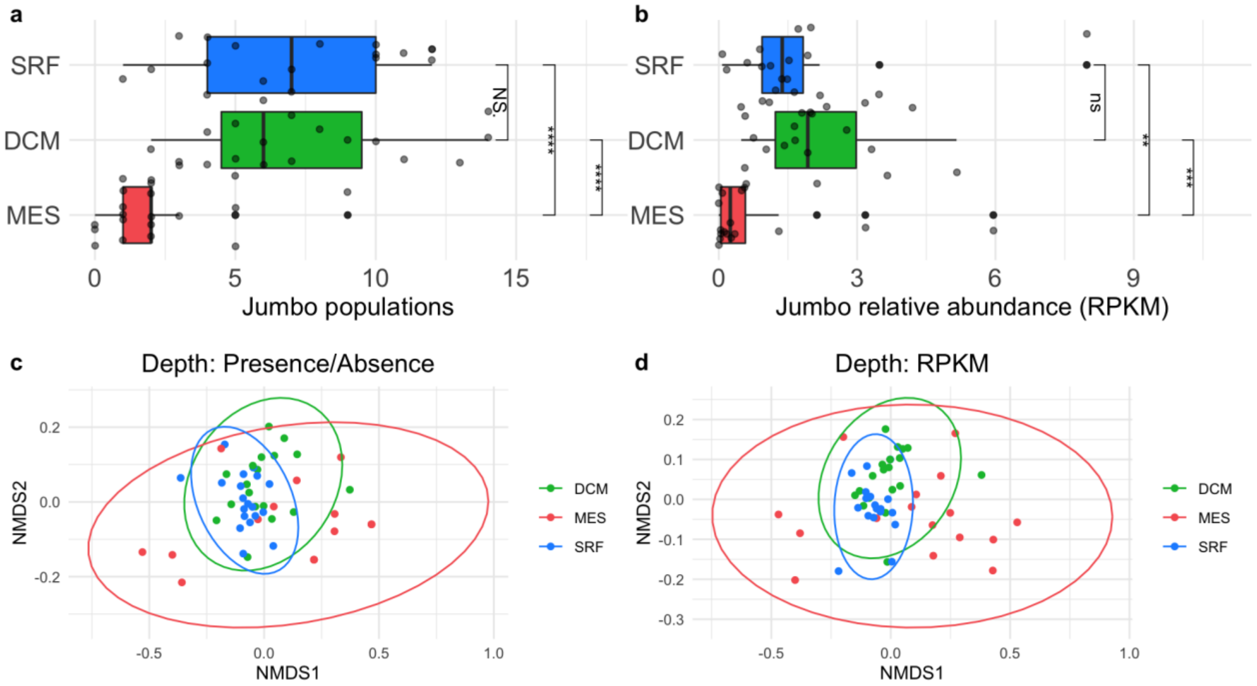

Supplemental Figure 9. (a,b) boxplots of jumbo populations present **(a)** and jumbo abundance in RPKM **(b)** in samples of stations co-collected at all three depths in the picoplankton fraction sorted by median abundance. Significance bars for a,b correspond to Wilcox test, with stars corresponding to p values < 0.05 and those with p values > 0.05 as not significant "ns" (ggplot2 stat_compare_means function). **(c,d)** NMDS plots of jumbo composition in those samples based on Bray-Curtis dissimilarity distances using jumbo populations' presence/absence data **(c)** and jumbo population relative abundance in RPKM **(d)** colored by depth. Green - DCM, red - MES, blue - SRF. Ellipses calculated by multivariate normal distribution. Depths were significantly different using ANOSIM (p values < 0.01).

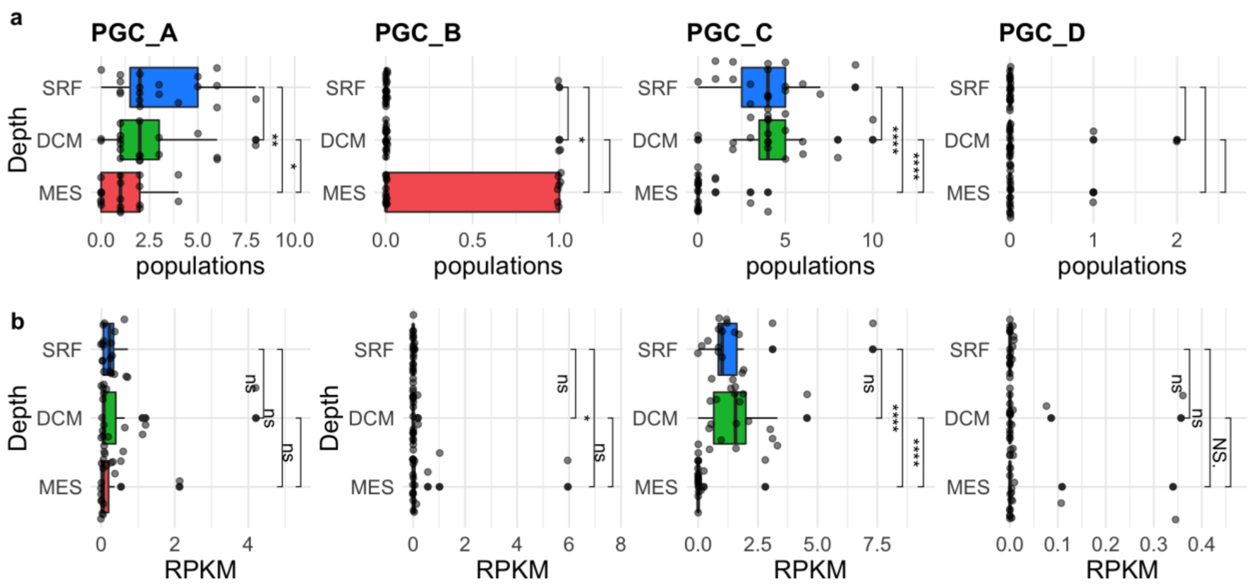

Supplemental Figure 10. (a,b) boxplots for PGCs A-D of jumbo populations present **(a)** and jumbo abundance in RPKM **(b)** in samples of stations co-collected at all three depths in the picoplankton fraction sorted by mean abundance. Significance bars correspond to Wilcox test, with stars corresponding to p value < 0.05 and those with p values > 0.05 as not significant "ns" (ggplot2 stat_compare_means function).

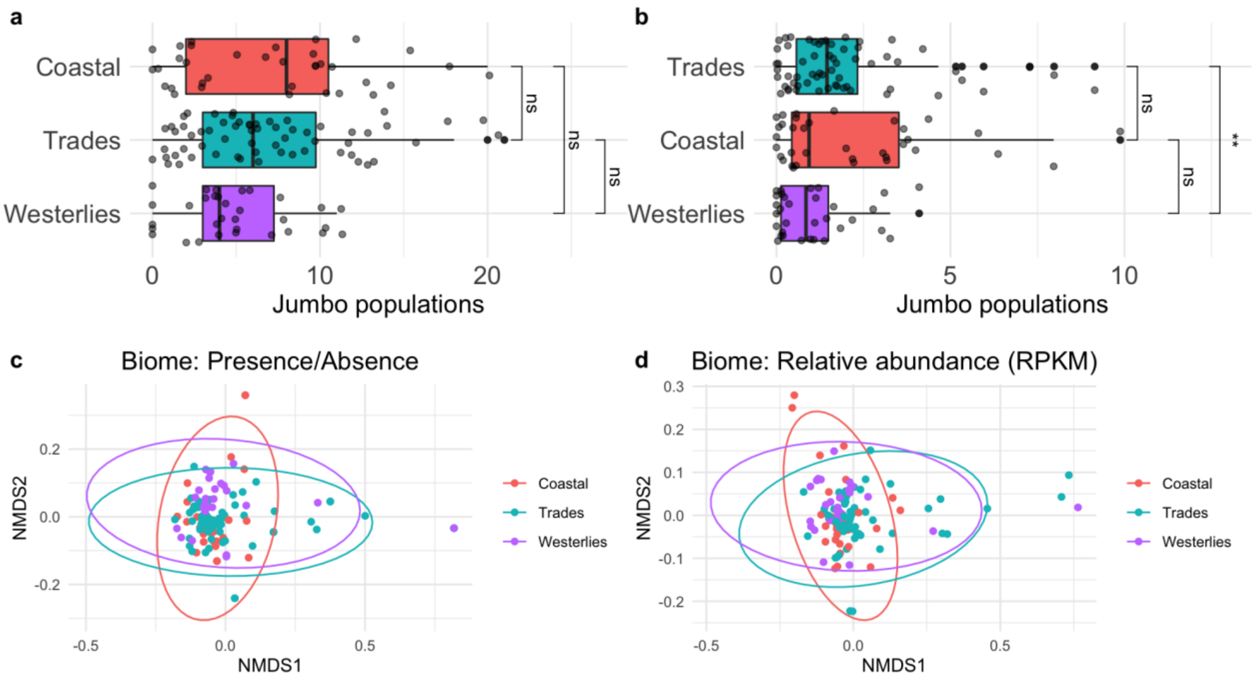

Supplemental Figure 11. (a,b) boxplots of jumbo abundance in RPKM **(a)** and jumbo population richness **(b)** in samples of the picoplankton fractions, sorted by median abundance at different biomes. **(c,d)** NMDS plots of jumbo composition in those samples based on jumbo

population abundance **(c)** and jumbo populations' presence **(d)** colored by biome. pink - Coastal, blue - Trades, purple - Westerlies. Ellipses calculated by multivariate normal distribution. Biomes were significantly different using ANOSIM (*p* values < 0.05). Significance bars in a,b correspond to Wilcox test, with stars corresponding to *p* values < 0.05 and those with *p* values > 0.05 as not significant "ns" (ggplot2 stat_compare_means function).

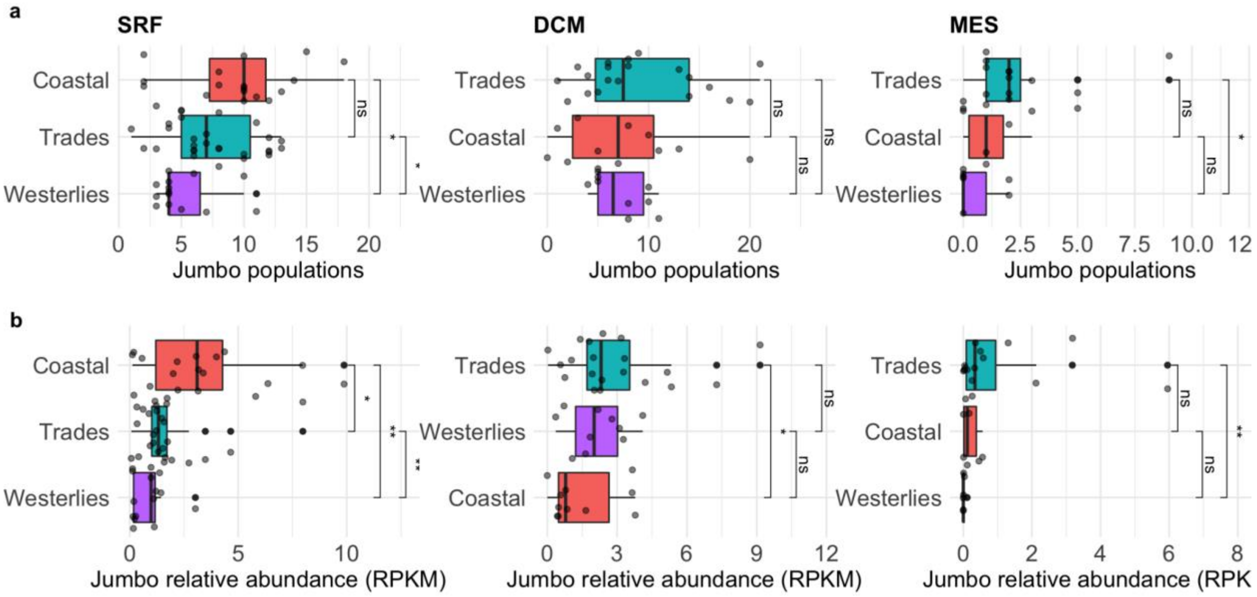

Supplemental Figure 12. (a,b) boxplots of jumbo abundance in RPKM **(a)** and jumbo population richness **(b)** in picoplankton samples of each depth separated by biome and sorted by median abundance in the different biomes. Significance bars correspond to Wilcox test, with stars corresponding to *p* values < 0.05 and those with *p* values > 0.05 as not significant "ns" (ggplot2 stat_compare_means function).

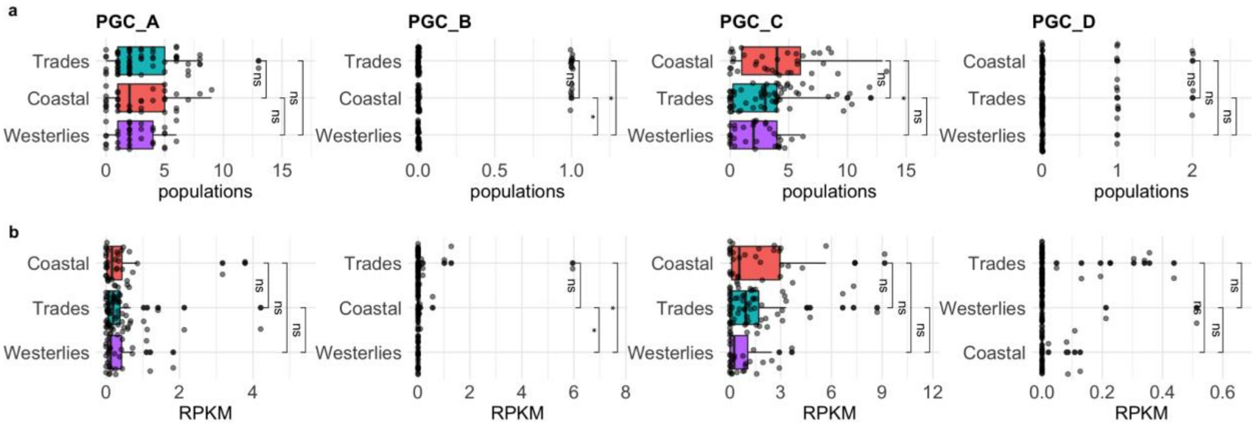

Supplemental Figure 13. (a,b) boxplots of jumbo abundance in RPKM **(a)** and jumbo population richness **(b)** in picoplankton samples of each cluster separated by biome and sorted by median abundance in the different biomes. Significance bars correspond to Wilcox test, with stars corresponding to *p* values < 0.05 and those with *p* values > 0.05 as not significant "ns" (ggplot2 stat_compare_means function).

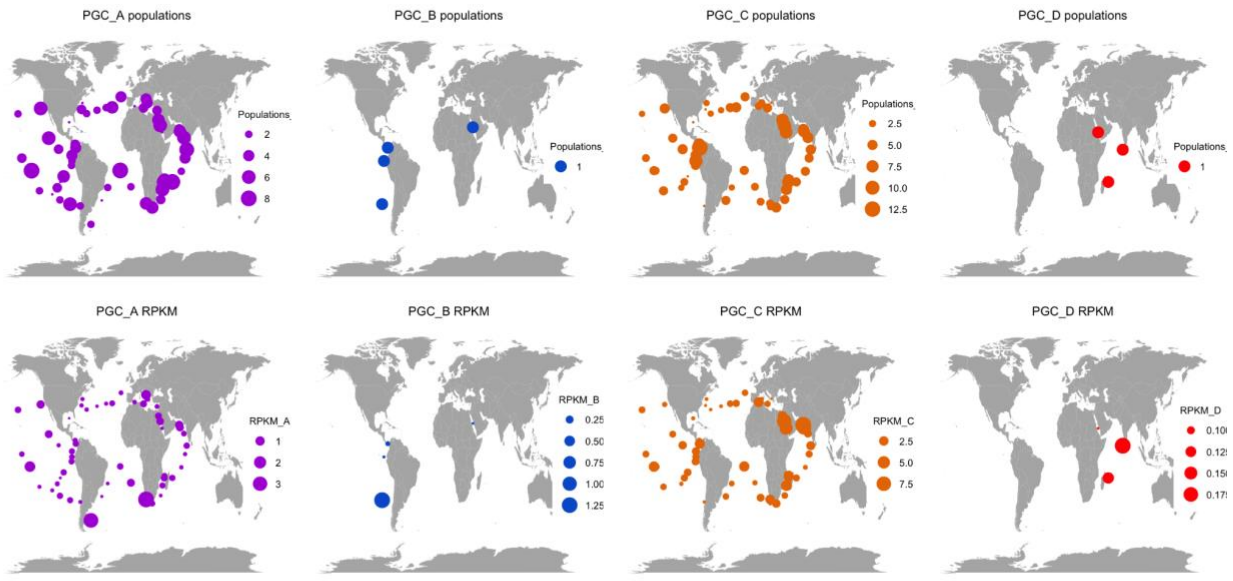

Supplemental Figure 14. Maps of the number of jumbo populations (top row maps) and total RPKM (bottom row maps) of jumbo phages in the titled PGC in surface samples of the picoplankton fraction colored by PGC.

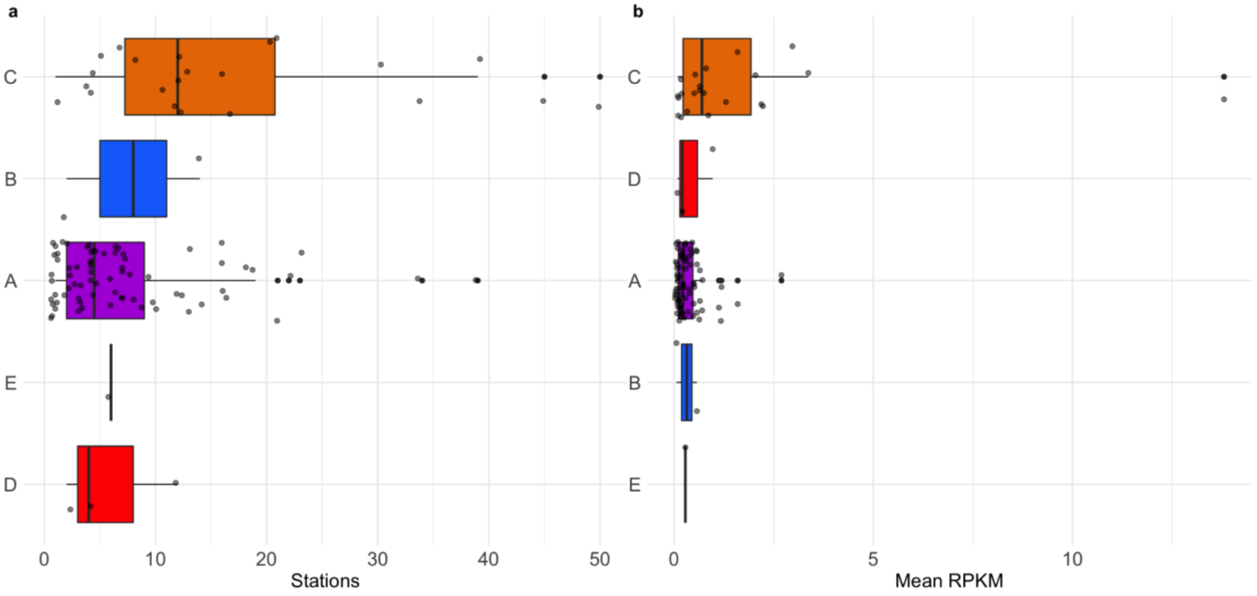

Supplemental Figure 15. Boxplots of the total number of stations a marine jumbo phage is present (a) and the mean RPKM that a jumbo phage is present (b) separated by PGC, sorted by median. Colors correspond to PGC.

Figures with viral fraction (<-0.22 or 0.1-0.22) results:

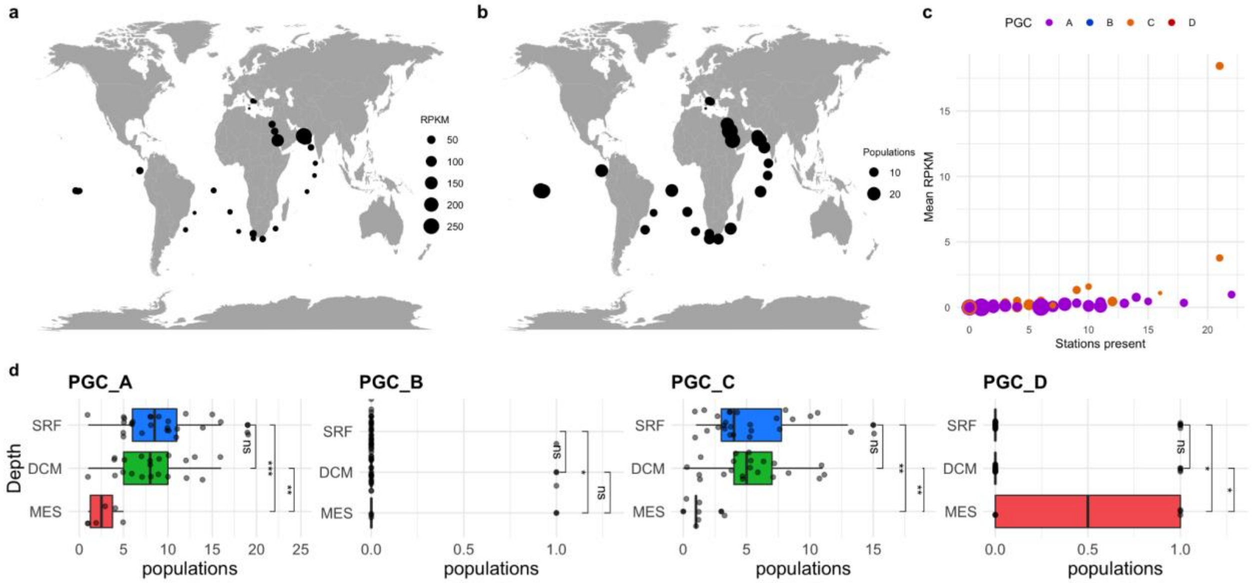

Figure 16. a,b) Maps of the relative abundance (**a**) of total jumbo phages (in RPKM) and (**b**) total number of jumbo populations present regardless of phage cluster membership in each surface (SRF) sample of the virome size fractions (either <-0.22 μm or 0.1-0.22 μm depending on availability). Dots sizes are proportional to the number of populations or RPKM and colored by biome (Coastal - pink, Westerlies - purple, Trades - blue). (**c**) Scatterplot of the mean RPKM of a jumbo population in SRF virome samples versus the number of SRF picoplankton stations it was present. Populations are colored by PGC and size corresponds to putative genome length in 100 kilobases. (**d**) Boxplot of the number of jumbo phage populations in a sample separated by depth sorted by median for each PGC. Significance bars correspond to Wilcox test, with stars corresponding to *p* values < 0.05 (stat_compare_means function)

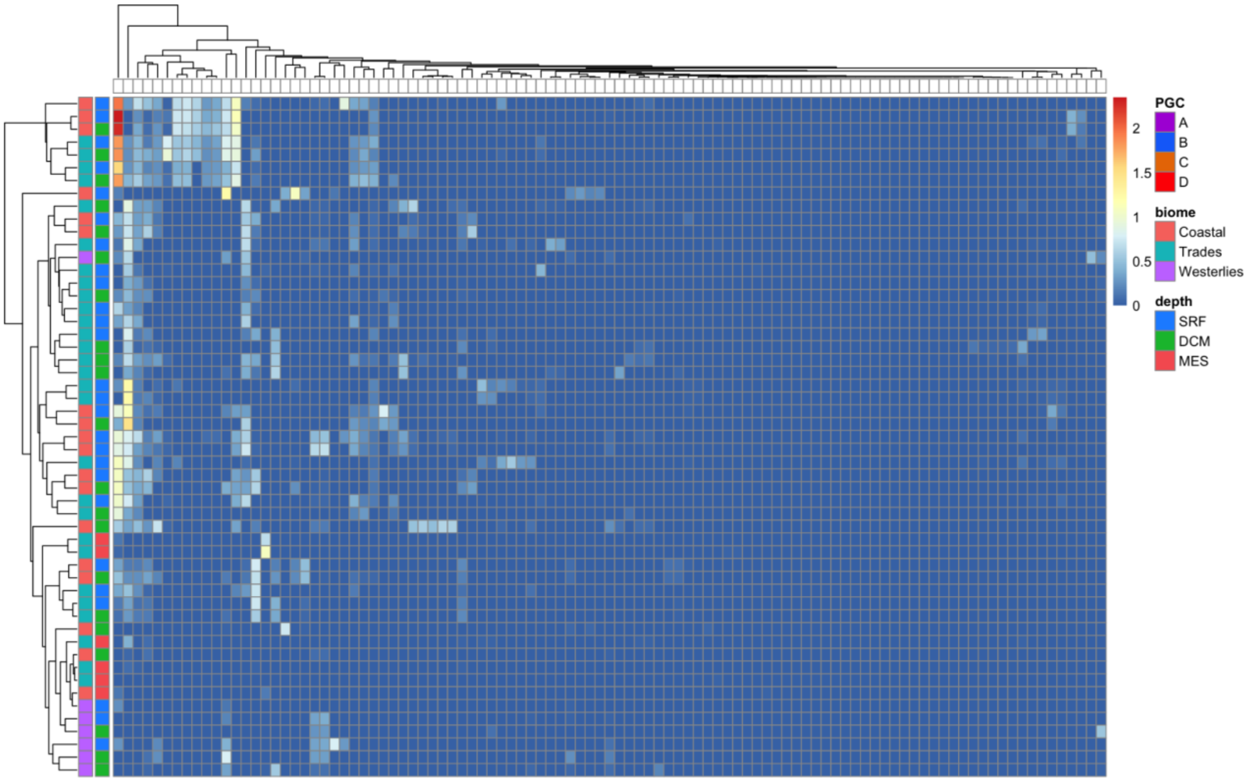

Supplemental Figure 17. Heat map of the log-transformed RPKM (log10(1+RPKM)) of each jumbo phage from this study (columns) in each picoplankton sample (rows). Rows and columns are clustered using hierarchical clustering via pheatmap default settings. Row annotation strip corresponds to each phage's PGC. The outer annotation strip on the columns corresponds to a sample's biome and the inner strip corresponds to a sample's depth.

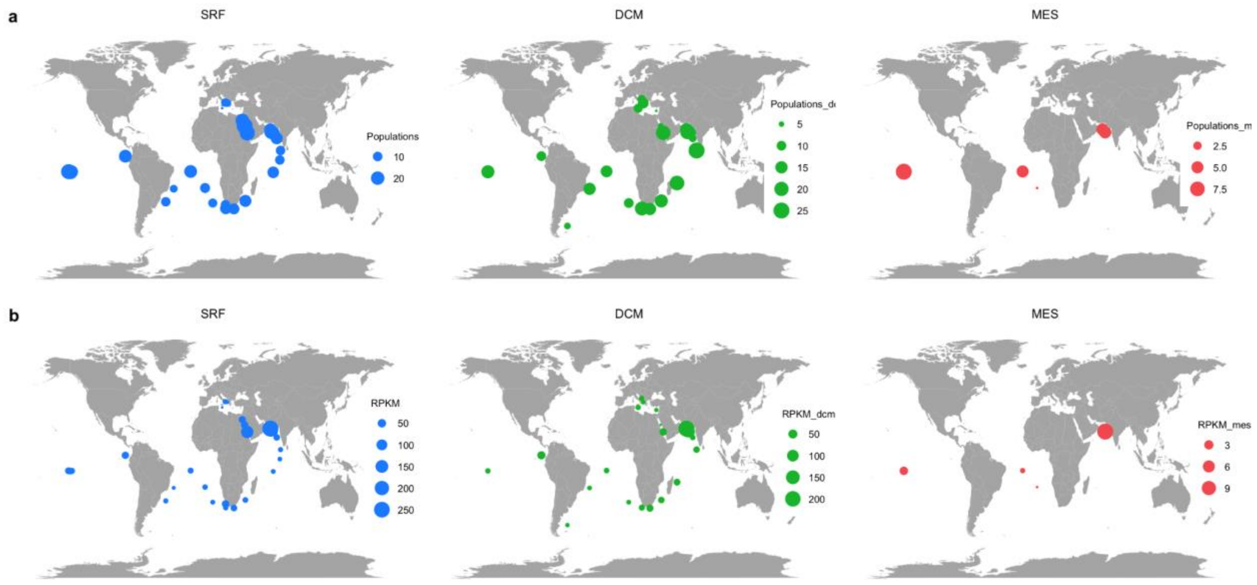

Supplemental Figure 18. Distribution of virome fractions (<-0.22 µm or 0.1-0.22 µm) at each depth. Points are colored by depth (SRF - blue, DCM - green, MES - red). Point sizes in upper row maps correspond to the number of jumbo populations in a sample and point sizes in bottom row maps correspond to jumbo relative abundance (RPKM).

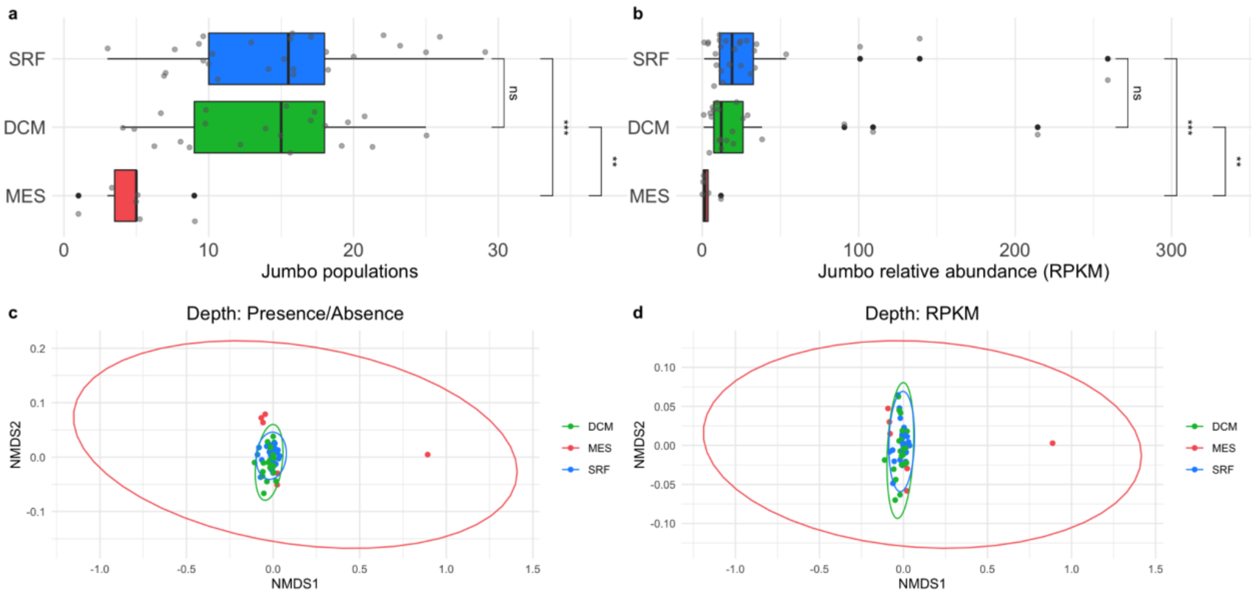

Supplemental Figure 19. (a,b) boxplots of jumbo populations present **(a)** and jumbo abundance in RPKM **(b)** in viral fraction samples by depth sorted by mean abundance. Significance bars for a,b correspond to Wilcox test, with stars corresponding to *p* values < 0.05 and those with *p* values > 0.05 as not significant "ns" (ggplot2 stat_compare_means function). **(c,d)** NMDS plots of jumbo composition in those samples based on Bray-Curtis dissimilarity distances using jumbo populations' presence/absence data **(c)** and jumbo population relative abundance in RPKM **(d)** colored by depth. Green - DCM, red - MES, blue - SRF. Ellipses

calculated by multivariate normal distribution. Depths were significantly different using ANOSIM (p values < 0.05).

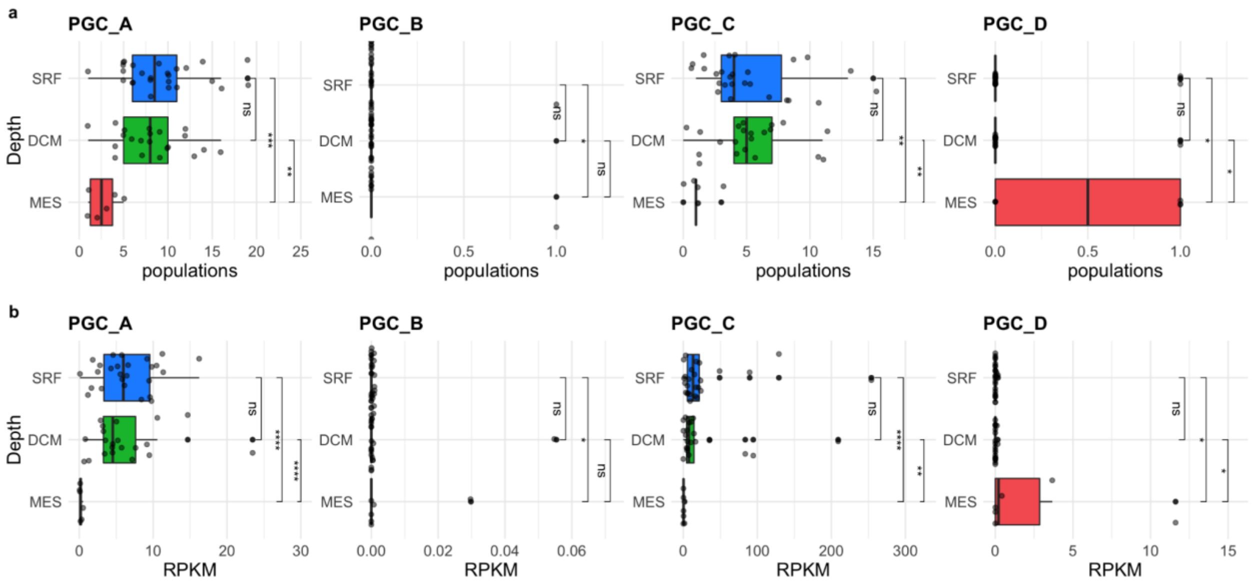

Supplemental Figure 20. (a,b) boxplots for PGCs A-D of jumbo populations present **(a)** and jumbo abundance in RPKM **(b)** in viral fractions samples of stations at all three depths sorted by mean abundance. Significance bars correspond to Wilcox test, with stars corresponding to p value < 0.05 and those with p values > 0.05 as not significant "ns" (ggplot2 stat_compare_means function).

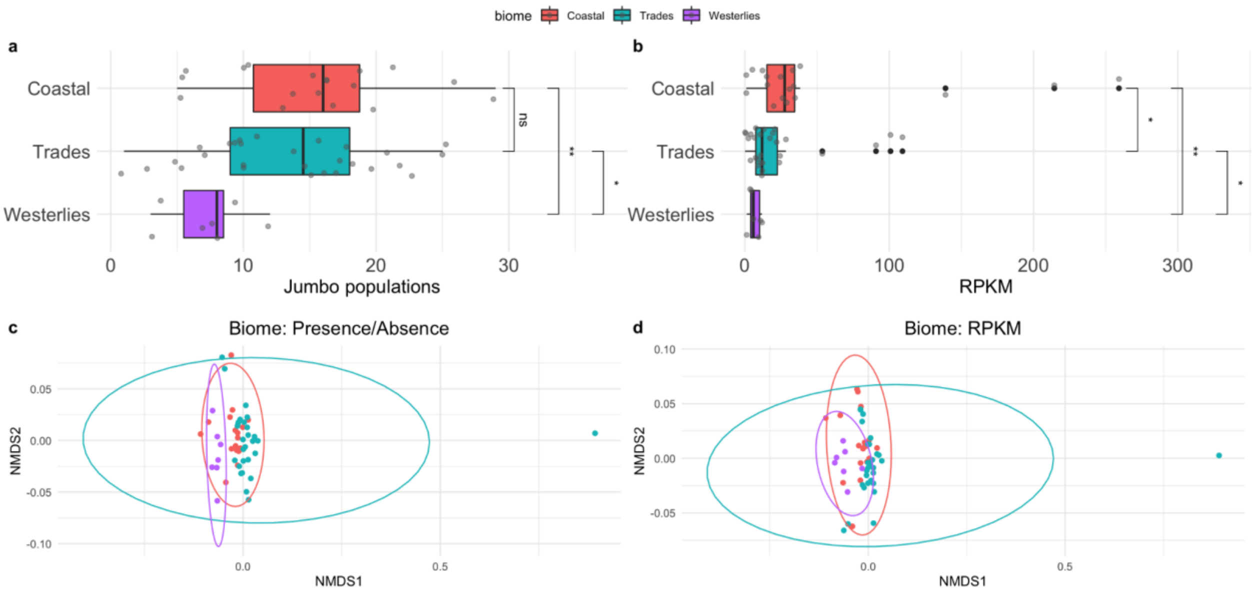

Supplemental Figure 21. (a,b) boxplots of jumbo abundance in RPKM **(a)** and jumbo population richness **(b)** in viral fraction samples sorted by median abundance at different biomes. **(c,d)** NMDS plots of jumbo composition in those samples based on jumbo population abundance **(c)** and jumbo populations' presence **(d)** colored by biome. pink - Coastal, blue -

Trades, purple - Westerlies. Ellipses calculated by multivariate normal distribution. Biomes were significantly different using ANOSIM (p value < 0.01, R statistic 0.2). Significance bars in a,b correspond to Wilcox test, with stars corresponding to p values < 0.05 and those with p values > 0.05 as not significant "ns" (ggplot2 stat_compare_means function).

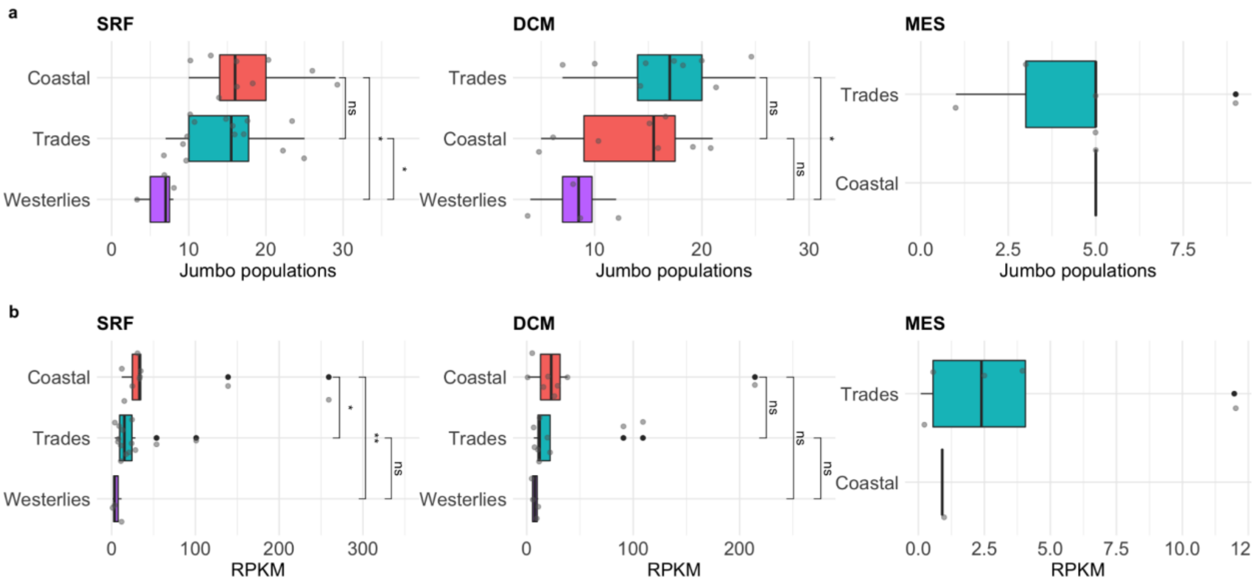

Supplemental Figure 22. (a,b) boxplots of jumbo abundance in RPKM **(a)** and jumbo population richness **(b)** in viral samples of each depth separated by biome and sorted by median abundance in the different biomes. Significance bars correspond to Wilcox test, with stars corresponding to p values < 0.05 and those with p values > 0.05 as not significant "ns" (ggplot2 stat_compare_means function).

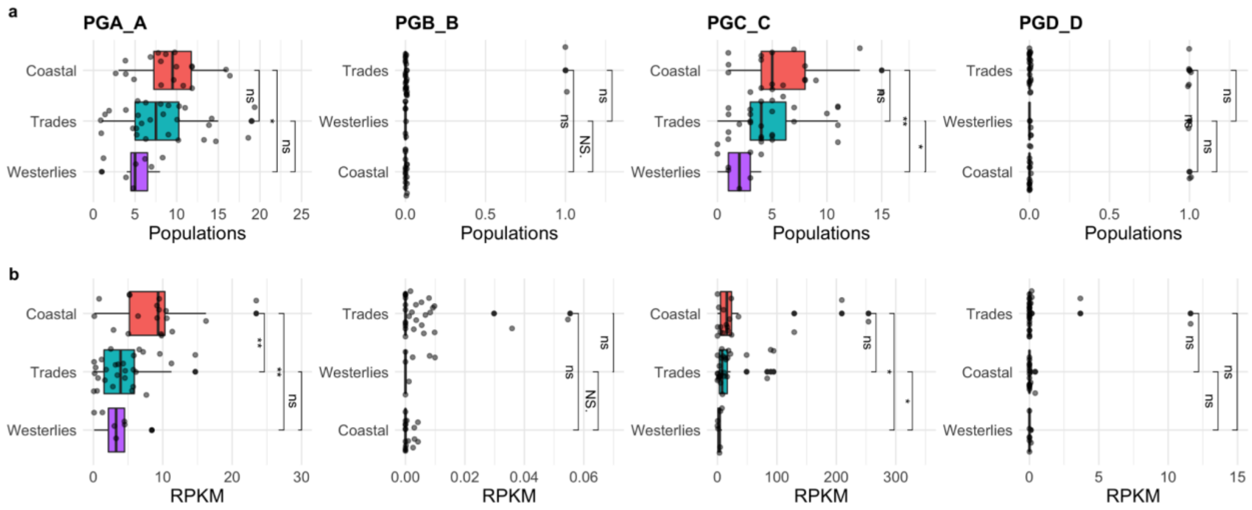

Supplemental Figure 23. (a,b) boxplots of jumbo abundance in RPKM **(a)** and jumbo population richness **(b)** in viral fraction samples of each cluster separated by biome and sorted by median abundance in the different biomes. Significance bars correspond to Wilcox test, with

stars corresponding to *p* values < 0.05 and those with *p* values > 0.05 as not significant "ns" (ggplot2 stat_compare_means function).

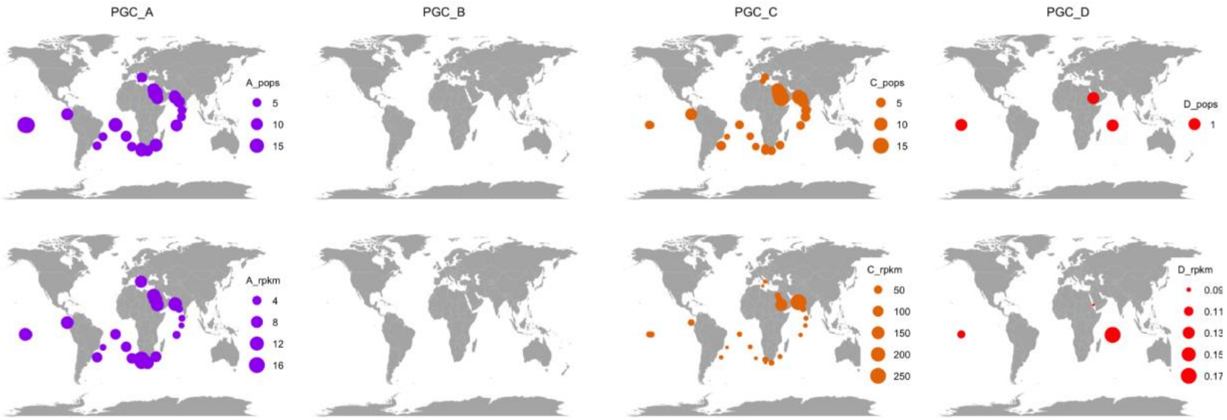

Supplemental Figure 24. Maps of the number of jumbo populations (top row maps) and total RPKM (bottom row maps) of jumbo phages in the titled PGC in surface samples of the viral fractions colored by PGC.

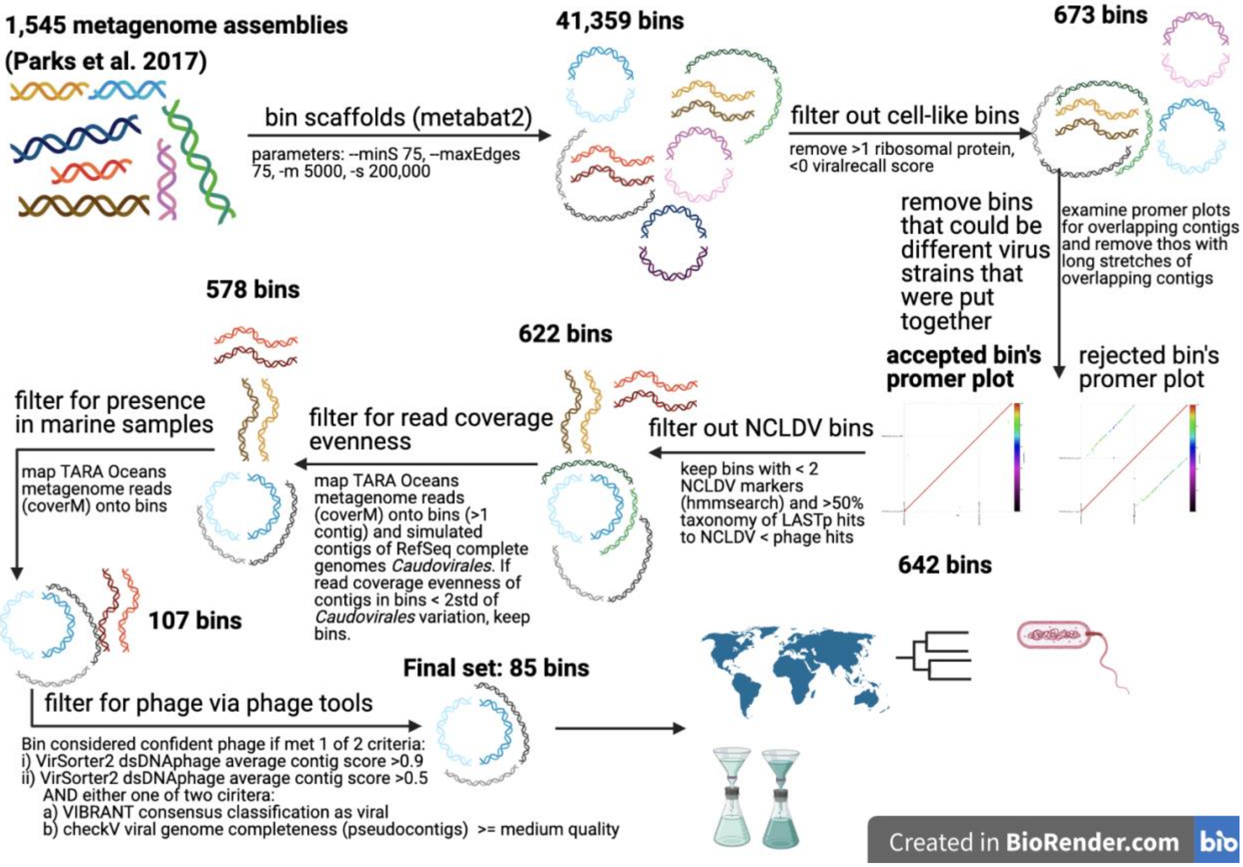

Supplemental Figure 25. Overview of binning pipeline. Details in Supplemental Methods.

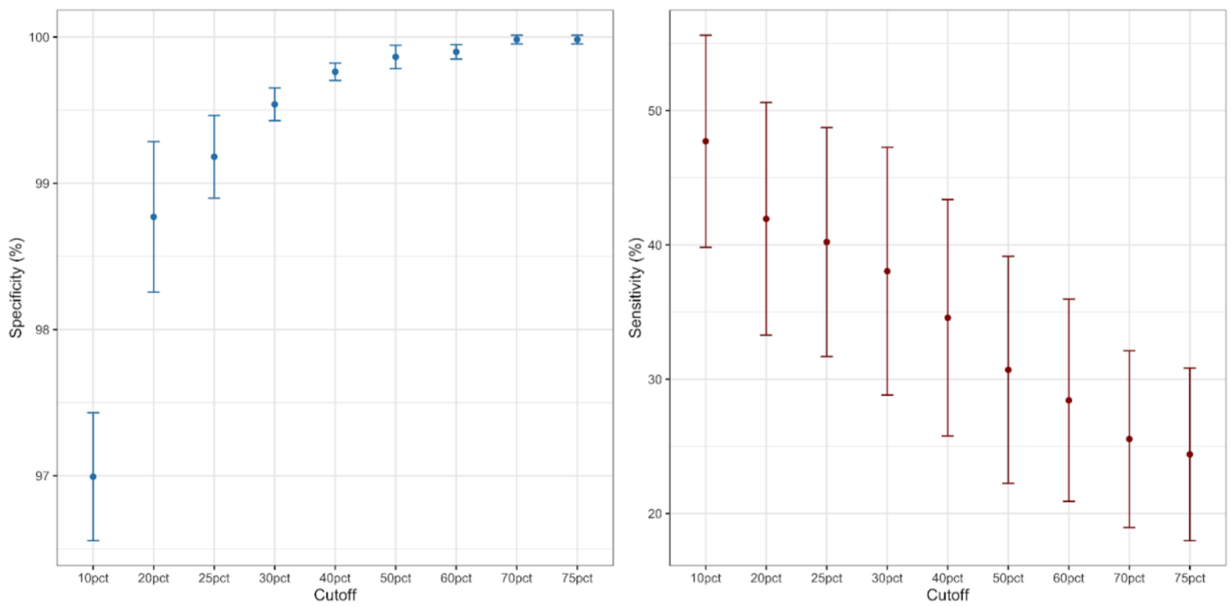

Supplemental Figure 26. Specificity (left) and sensitivity (right) of reference phage identification in simulated metagenomic communities (Roux et al. 2017). Different % covered fraction thresholds are shown on the x-axis. Error bars denote standard error.

Supplemental Methods with References

Supplemental Methods

Binning and screening for non-phage bins.

Contig sequences and coverage information from 1,545 metagenomes were downloaded from Parks et al 2017 (Parks et al. 2017). Contigs were binned with MetaBAT 2 (Kang et al. 2019) with the options --maxEdge 75 --minS 75 -m 5000, and -s 200000, which resulted in 41,359 bins.

Bins were then filtered for containing a maximum of 5 contigs (1,456 bins remained). We then predicted the proteins on each bin with prodigal (Hyatt et al. 2010) using default options. To begin filtering out bins potentially belonging to cells, we removed bins that encoded more than 1 ribosomal protein, which were detected with hidden markov model (HMM) searches via HMMER version 3.2.1) (Eddy 2011) (E value 0.001) against 27 Cluster of Orthologous Groups ribosome protein HMM profiles (Galperin et al. 2021) (1,043 bins remained). Next, we ran a beta version of ViralRecall (Moniruzzaman et al. 2020) on the bins to remove bins that had negative scores, which indicate they encode more cellular proteins than viral proteins (673 bins remained).

To address the automated binning complication of strain heterogeneity (cases where contigs binned together based on similar tetranucleotide frequencies and coverage, but actually belong to different viruses), we examined for potential overlapping of conserved regions between contigs by running promer (--maxmatch option) via MUMmer (Kurtz et al. 2004), which compares sequences to each other. We then examined this output with mummerplot (--color --png options) for cases where contigs contained extended conserved regions with other contigs in the bin and discarded these bins (example in Supplemental Figure 15).

The remaining 642 bins were then screened for Nucleocytoplasmic Large DNA Viruses (NCLDV) by searching the bin proteins for 8 NCLDV markers with an HMM search (E value 0.001). These eight markers included the following with the minimum bitscore cutoff to be considered a hit in parentheses: A32 (200), D5 (200), SFII (200), mcp (200), mRNAc (200), PolB (500), RNR (200), VLTF3 (200). Additionally, a LASTp (Kiełbasa et al. 2011) was run on the proteins of the 642 bins against RefSeq r99 (E value < 0.001). If the taxonomy of hits to phage proteins outnumbered hits to NCLDV proteins or the number of NCLDV markers was below 2, the bin was considered phage (622 bins remained). Bins were then filtered to remove spurious contigs by removing bins with contigs shorter than 5 kb (610 bins remained). Additionally, we removed bins that contained potentially contaminating contigs based on read mapping coverage (see below).

Validation of bins with multiple metagenomic read mapping and detection in marine samples
To further ensure contigs belonging to different phages were not spuriously binned together, we assessed for evenness in contig coverage by mapping reads from different metagenomes to the bins. We used Tara Oceans metagenomes for the mapping (Sunagawa et al. 2015) so these results could also be used to detect marine jumbo phages. Specifically, we focused on results from samples filtered above 0.22 μm to minimize instances of fragmented capsids or free DNA complicating coverage results, which may be more likely in the viral fractions if a capsid is larger than 0.22 μm. Because read mapping evenness can vary in phage genomes due to conserved regions (Sieradzki et al. 2019), we used mapping results from a reference dataset to benchmark a threshold variation level. For this, we compiled this reference dataset by downloading nucleotide sequences of all complete genomes belonging to the *Caudovirales* order on NCBI's Viral Genomes Portal on July 5, 2020 (referred to as "RefSeq Caudo") and subsetted for jumbo phages ("RefSeq jumbo"); we also included jumbo phage sequences curated by Al-Shayeb et al 2020 (Al-Shayeb et al. 2020). We fragmented these reference jumbo reference sequences with an in-house python script into contigs (1-5) of over 10 kb in length. We then mapped the Tara Oceans metagenomes to this reference set and the bins with multiple contigs (342 bins) with coverM (wwood n.d.) (coverm contig --min-read-percent-identity 95 -m covered_fraction rpkm count variance length -t 32 --minimap2-reference-is-index --coupled; database of phages for mapping was created with minimap2 minimap2 -x sr -d)(Li 2021) and retained phages with at least 10% covered. Next, we calculated the standard deviation of coverage reported in reads per kilobase per million (RPKM) of the different contigs in a bin with a python script. Reads per kilobase per million is calculated by dividing the number of reads mapped to a sequence by the sequence length in kilobases to account for differences in sequence length between genomes and then dividing that by the million number of reads in the sample to account for differences in read depth between samples. The RPKM tables of the bins and references were split by Tara Oceans depth ("env") type (SRF, DCM, MES). In R (3.5.1) (R Core Team 2019) via RStudio (1.1.456). For each depth, we set the maximum standard deviation cutoff to the 95th percentile standard deviation value of the reference RPKM variation (i.e. quantile(reference_srf$std_dev, 0.95). To determine the percentage of samples a bin must have mapped below the reference 0.95 cutoff at each depth, we filtered the bin RPKM table for each depth using percent below the cutoff until the distribution of the standard deviation values for the bins was not significantly different from the reference distribution using a Wilcox test (*p* value > 0.05). 310 of the 342 bins

passed. 268 bins comprised only one contig, totaling the bins at 578. Based on the read mapping results from samples of all size fractions, a jumbo phage was considered present in a sample if at least 10% of its genome was mapped by the sample. Of the 578 bins that passed the validation test, 107 bins were present in marine samples.

Validation of bins as phage with phage-detection tools and population clustering with other jumbo phages

Contigs of the remaining 107 bins were run through VirSorter2 (Guo et al. 2021), VIBRANT (Kieft, Zhou, and Anantharaman 2020), and CheckV (Nayfach et al. 2021). CheckV was also run on pseudocontigs of multi-contig bins that were generated using an in-house python script to join the contigs of the bins together with "N"s. First, bins were retained if the VirSorter2 dsDNAphage score of their contigs averaged above 0.9 (75 bins). Next, bins were retained that had a minimum VirSorter2 dsDNA score average above 0.5 and either had been (i) classified as "virus" by VIBRANT or (ii) considered viral by CheckV with genome quality of medium or above. To further ensure the bins contained non-redundancy between their contigs or contamination, we ran CheckV on the bin's contigs individually and examined the completeness estimation. Only 4 contigs were detected as complete, circular genomes (3 high confidence, 1 low confidence) based on direct terminal repeats (DTRs). These contigs belonged to bins that only contained one contig, suggesting these single-contig bins represent complete jumbo phage genomes. The bins used for subsequent analyses then totaled at 85 bins.

Prior to further gene-based analyses, we checked if the bins of jumbo phages used alternative genetic codes, as has been found for some jumbo phages(Devoto et al. 2019; Al-Shayeb et al. 2020), with Codetta(Shulgina and Eddy 2021) (default options). None clearly used codes other than the standard code 11, and we proceeded with the initial prodigal protein predictions.

We then compared the bins to other jumbo phages and identified those belonging to the same population, defined by sharing over 80% of genes with at least 95% average nucleotide identity with one or more other phages in the population (single-linkage)(Brum et al. 2015). This jumbo phage reference set included those on RefSeq belonging to the *Caudovirales* (93 phages), those prepared by Al-Shayeb et al. 2020 (336 phages) (Al-Shayeb et al. 2020), those available in GenBank compiled by Iyer et al 2021 (Iyer et al. 2021) and Cook et al 2021(Cook et al., n.d.) (400 phages), GOV 2.0 (60 phages) (Gregory et al. 2019), ALOHA 2 (8 phages) (Luo et al. 2020), and one megaphage from the English Channel (Michniewski et al. 2021). These additional jumbo phage sequences and the phage sequences from RefSeq jumbo are referred to as the "jumbo references", totaled at 898 sequences. Nucleotide and amino acid sequences of genes encoded by the 85 jumbo bins and the 898 jumbo references were predicted with prodigal using the default genome setting for each genome individually (-a,-d options). These genes were then aligned to each other with BLASTn. Bins were considered belonging to the same population if 80% of their genes aligned to another bin's genes with an average nucleotide identity of at least 95% (Brum et al. 2015). This analysis resulted in 535 jumbo phage populations, 59 of which contained a jumbo bin generated from this study and 47 populations solely contained bins from this study.

Bipartite network analysis

Jumbo bins were clustered with the jumbo references and *Caudovirales* on RefSeq of all genome sizes and reference jumbo phage set described above based on composition Virus Orthologous Groups (VOG: vogdb.org, downloaded April 14, 2020). Amino acid sequences were searched against HMM profiles in the VOG database via HMM searches (E-value < 0.001). A matrix of VOG families as columns and phage as rows was generated from the hmm output with an in-house python script (Supplemental Dataset 2). The matrix was loaded into R and an incidence graph was computed with the R library igraph(1.2.5) ("Igraph – Network Analysis Software" n.d.). Clusters were then detected with the spinglass algorithm (Reichardt and Bornholdt 2006) using 50 spins. The spinglass clustering was run 100 times with different seeds. The final clusters were discerned based on the iteration that yielded the highest modularity (seed 544, modularity 0.5856642). Network was visualized with igraph using the Fruchterman-Reingold layout with 5000 iterations (layout.fruchterman.reingold(niter=5000)). Clusters of which the jumbo bins belonged were plotted with ggplot2(3.1.1) (Wickham 2011a) in R for composition of RefSeq phages host phyla and dataset origin; figures were joined with ggpubr(0.2.4) and Inkscape(v 0.92).

MCP and TerL Phylogenies

All major capsid protein (MCP) and terminase large subunit HMM profiles were compiled from vogdb.org (release 98) (see FigShare (https://figshare.com/account/home#/projects/127391)). Proteins of the jumbo bins, jumbo references, and all other *Caudovirales* on RefSeq were searched against these databases with HMM searches (-E 0.001 flag). To reduce the dataset to facilitate phylogenetic analyses and improve the alignment quality, we took only the best hit (highest bitscore) encoded by a phage and then removed protein sequences that were less than two standard deviations below the median length encoded by the references, which was 96 amino acids (aa) for the MCP and 170 aa for TerL. This quality filtering resulted in 74 MCP protein sequences encoded by the bins, 3,193 MCP proteins encoded by the references, 80 TerL proteins encoded by the bins, and 3,466 TerL proteins encoded by the references. To further reduce the reference dataset, we clustered the reference hits with cd-hit(Fu et al. 2012) using a 90% ID cutoff (-c 0.9 option). This clustering resulted in 1,180 reference MCP and 1,348 reference TerL protein sequences.

In total, 1,254 MCP protein sequences and 1,428 TerL sequences were aligned separately with Clustal Omega (Sievers et al. 2011). The alignments were then trimmed using trimAl (parameter -gt 0.1) (Capella-Gutiérrez, Silla-Martínez, and Gabaldón 2009). A tree was reconstructed with this alignment using IQ-TREE (Nguyen et al. 2015) with ModelFinder (Kalyaanamoorthy et al. 2017) to select the best fit model according to the Bayesian Information Criterion, which was VT+F+G4 for the MCP alignment and Blosum62+F+G4 for the TerL alignment and 1,000 ultrafast bootstrap replicates. Trees were visualized in iTOL (v5) (Letunic and Bork 2021) and jumbo bins were colored with network cluster. Figures were joined with ggpubr and Inkscape.

Annotation

Amino acid sequences of jumbo phages were searched with HMM searches against HMM profiles of the EggNOG 5.0(Huerta-Cepas et al. 2019) (E-value <0.001), VOG (release 98), and Pfam (Pfam-A, version 32) (Mistry et al. 2020) databases. To identify virion structural proteins,

structural proteins in VOG were manually identified (Supplemental Dataset 3). A consensus annotation of a protein was determined based first by Pfam hit because these functions are well-curated (Mistry et al. 2020), then by VOG or EggNOG hit based on bitscore. Pfam does not assign functional categories, so a gene's functional category was based on the category of its EggNOG hit or virion structure designation. These categories were subsequently merged into broader categories (Supplementary Dataset 3). Functions that had multiple EggNOG categories (i.e. NK) were tallied individually. Stacked barplots of the functional composition of each jumbo phage cluster were based on the average proportion of genes belonging to the category and plotted in R with ggplot2. Genes with known functions that drove variation between clusters A-D of this study were identified by first calculating the proportion of genomes which encoded a given gene in each cluster and then calculating the variance of the proportion of genomes among the clusters in R. Genes with variance above 0.2 and known functions were retained for heatmap visualization made with pheatmap in R.

Group 1.0 jumbo phages, belonging to PGC_B in this study, are known to encode a divergent family B DNA polymerase. As this gene has not been included in the databases examined, we identified the HMM profile of the VOG family corresponding to this divergent family B DNA polymerase by searching a reference sequence for this gene (YP_009153312.1) with an HMM search (-E 0.001) against the VOG database, which was VOG09941 (bitscore > 1000). We then compared the bitscore of genes that hit to this VOG with the classic family B DNA polymerase (PF00136) to identify the occurrence of the divergent family B DNA polymerase in the phages.

Distribution analyses

To examine the distribution of populations of jumbo phages in the ocean, we mapped reads from the Tara Oceans metagenomes used in the bin validation, but excluded Polar samples as there were only 5 available in this set. Reads were trimmed and subsampled to 20 million per sample. They were then mapped onto the representative sequences of the 535 jumbo phage populations as follows. The reference database of the representative jumbo phage sequences was created with minimap2 (minimap2 -x sr -d) and the mapping was carried out with on the jumbo phages with coverM (coverm genome --min-read-percent-identity 95 -m covered_fraction rpkm count variance length -t 32 --minimap2-reference-is-index --coupled); Mapping results were retained if at least 20% of the phage genome was covered (see *Benchmarking percent coverage for distribution* section below this section). To compare mapping results between phages and samples, reads per kilobase per million (RPKM) was then calculated by dividing the number of reads aligned to a phage by the length of the phage in kilobases and then dividing that by the number of reads in the sample in millions, which accounts for differences in phage sequence length and difference in sample sequencing depth. Statistical tests and plots of the mapping results were carried out in R with the stat_compare_means(label="p.signif") function in ggplot2 to compare samples between biomes, fractions, and depth in richness and abundance of jumbo phage populations. Compositional differences of jumbo phage between samples based on both presence/abundance and RPKM matrices were compared with ANOSIMs in R using the anosim function from the package vegan(2.5-5) (Dixon 2003) (distance="bray",permutations=9999). Maps were plotted in R with the maps (“Maps: Draw Geographical Maps” n.d.) and ggplot2 libraries. Boxplots were plotted in R with ggplot2 and

plyr(1.8.4) (Wickham 2011b) to order axes. Non-metric dimensional scaling plots were generated in R based on Bray Curtis dissimilarity matrices (vegdist (method="bray)) using the metaMDS vegan function and visualized with ggplot2; ellipses were calculated with stat_ellipse(type="norm"). Figures were joined with ggpubr in R.

Benchmarking percent coverage for distribution

We considered a jumbo phage to be present in a sample if at least 20% of the genome could be recovered by read mapping with at least 1X coverage (a 20% fraction covered cutoff). To ensure that this was an appropriate cutoff that did not lead to a large number of false-positive identifications, we benchmarked different cutoffs using three *in silico* viromes generated in a previous study (Roux et al. 2017). We downloaded the trimmed reads from the mock communities labelled Samples 1, 2, and 3 (10 million paired-end reads per sample) and mapped the reads against the complete reference genomes that were used in their construction. The databases used for mapping also included ~2,000 *Caudovirales* genomes selected from the INPHARED database that were added to assess the incidence of false positive phage detection (Supplemental Dataset 4). The additional genomes were selected randomly from a set of *Caudovirales* in INPHARED that had a MASH (Ondov et al. 2016) distances >0.05 when compared to all genomes used to make the mock communities; this was done because the addition of genomes that were closely-related to those used to make the mock communities cannot be considered to be true false positives. We mapped reads with CoverM using the same parameters we used in our jumbo phage work (95% identity), and we then calculated the sensitivity and specificity of different % fraction covered cutoffs (see Supplemental Figure 26). These results revealed that a 20% fraction covered cutoff had a specificity >98%, indicating that it is appropriate for our purposes and that higher values would further decrease sensitivity without a marked increase in specificity.

Host prediction

Hosts were estimated for the bins based on CRISPR spacers, tRNAs, and the taxonomy of genes. CRISPR spacers were predicted for the Genome Taxonomy Database (release 95)(Parks et al. 2018) and metagenome assembled genomes (MAGs) of bacteria and archaea from the metagenomes of which the jumbo phages derived by Parks et al 2017 (Parks et al. 2017), as well of the jumbo bins. All spacers were aligned to the jumbo bins and hits were at least 24 basepairs in length with <= 1 mismatch (Al-Shayeb et al. 2020) via BLASTn (-task blastn-short). No hosts could be assigned with this approach, and no jumbo bins targeted other jumbo bins. tRNAs were predicted on the jumbo bins and the same MAGs set with tRNAscan-SE (Lowe and Chan 2016) (-bacteria option). Promiscuous tRNAs were downloaded from Paez-Espino et al. 2016(Paez-Espino et al. 2016) and removed based on BLASTn hits (100% ID, <= 1 mismatches). Jumbo tRNAs were then aligned against the MAGs tRNAs with BLASTn and matches were considered with 100% ID and no more than one mismatch. Jumbo phage tRNA sequences were also searched against NCBI's nonredundant database using the BLASTn webserver and matches were retained with the same criteria. Finally, hosts were assigned based on the taxonomy BLASTn hits to the coding sequences of the MAGs. A putative host phylum was considered if a phylum had three times as many hits than the phylum with the next most hits (Al-Shayeb et al. 2020).

References

Al-Shayeb, Basem, Rohan Sachdeva, Lin-Xing Chen, Fred Ward, Patrick Munk, Audra Devoto, Cindy J. Castelle, et al. 2020. “Clades of Huge Phages from across Earth’s Ecosystems.” *Nature* 578 (7795): 425–31.

Brum, Jennifer R., J. Cesar Ignacio-Espinoza, Simon Roux, Guilhem Doulcier, Silvia G. Acinas, Adriana Alberti, Samuel Chaffron, et al. 2015. “Ocean Plankton. Patterns and Ecological Drivers of Ocean Viral Communities.” *Science* 348 (6237): 1261498.

Capella-Gutiérrez, Salvador, José M. Silla-Martínez, and Toni Gabaldón. 2009. “trimAl: A Tool for Automated Alignment Trimming in Large-Scale Phylogenetic Analyses.” *Bioinformatics* 25 (15): 1972–73.

Cook, Ryan, Nathan Brown, Tamsin Redgwell, Branko Rihtman, Megan Barnes, Dov J. Stekel, Martha Clokie, Jon Hobman, Michael Jones, and Andrew D. Millard. n.d. “INfrastructure for a PHAge REference Database: Identification of Large-Scale Biases in the Current Collection of Phage Genomes.” https://doi.org/10.1101/2021.05.01.442102.

Devoto, Audra E., Joanne M. Santini, Matthew R. Olm, Karthik Anantharaman, Patrick Munk, Jenny Tung, Elizabeth A. Archie, et al. 2019. “Megaphages Infect Prevotella and Variants Are Widespread in Gut Microbiomes.” *Nature Microbiology* 4 (4): 693–700.

Dixon, Philip. 2003. “VEGAN, a Package of R Functions for Community Ecology.” *Journal of Vegetation Science*. https://doi.org/10.1111/j.1654-1103.2003.tb02228.x.

Eddy, Sean R. 2011. “Accelerated Profile HMM Searches.” *PLoS Computational Biology* 7 (10): e1002195.

Fu, Limin, Beifang Niu, Zhengwei Zhu, Sitao Wu, and Weizhong Li. 2012. “CD-HIT: Accelerated for Clustering the next-Generation Sequencing Data.” *Bioinformatics* 28 (23): 3150–52.

Galperin, Michael Y., Yuri I. Wolf, Kira S. Makarova, Roberto Vera Alvarez, David Landsman, and Eugene V. Koonin. 2021. “COG Database Update: Focus on Microbial Diversity, Model Organisms, and Widespread Pathogens.” *Nucleic Acids Research*. https://doi.org/10.1093/nar/gkaa1018.

Gregory, Ann C., Ahmed A. Zayed, Nádia Conceição-Neto, Ben Temperton, Ben Bolduc, Adriana Alberti, Mathieu Ardyna, et al. 2019. “Marine DNA Viral Macro- and Microdiversity from Pole to Pole.” *Cell* 177 (5): 1109–23.e14.

Guo, Jiarong, Ben Bolduc, Ahmed A. Zayed, Arvind Varsani, Guillermo Dominguez-Huerta, Tom O. Delmont, Akbar Adjie Pratama, et al. 2021. “VirSorter2: A Multi-Classifier, Expert-Guided Approach to Detect Diverse DNA and RNA Viruses.” *Microbiome* 9 (1): 37.

Huerta-Cepas, Jaime, Damian Szklarczyk, Davide Heller, Ana Hernández-Plaza, Sofia K. Forslund, Helen Cook, Daniel R. Mende, et al. 2019. “eggNOG 5.0: A Hierarchical, Functionally and Phylogenetically Annotated Orthology Resource Based on 5090 Organisms and 2502 Viruses.” *Nucleic Acids Research* 47 (D1): D309–14.

Hyatt, Doug, Gwo-Liang Chen, Philip F. Locascio, Miriam L. Land, Frank W. Larimer, and Loren J. Hauser. 2010. “Prodigal: Prokaryotic Gene Recognition and Translation Initiation Site Identification.” *BMC Bioinformatics* 11 (March): 119.

“Igraph – Network Analysis Software.” n.d. Accessed July 23, 2021. http://igraph.org.

Iyer, Lakshminarayan M., Vivek Anantharaman, Arunkumar Krishnan, A. Maxwell Burroughs, and L. Aravind. 2021. “Jumbo Phages: A Comparative Genomic Overview of Core Functions and Adaptions for Biological Conflicts.” *Viruses* https://doi.org/10.3390/v13010063.

Kalyaanamoorthy, Subha, Bui Quang Minh, Thomas K. F. Wong, Arndt von Haeseler, and Lars S. Jermiin. 2017. “ModelFinder: Fast Model Selection for Accurate Phylogenetic Estimates.” *Nature Methods* 14 (6): 587–89.

Kang, Dongwan D., Feng Li, Edward Kirton, Ashleigh Thomas, Rob Egan, Hong An, and Zhong Wang. 2019. “MetaBAT 2: An Adaptive Binning Algorithm for Robust and Efficient Genome Reconstruction from Metagenome Assemblies.” *PeerJ* 7 (July): e7359.
Kieft, Kristopher, Zhichao Zhou, and Karthik Anantharaman. 2020. “VIBRANT: Automated Recovery, Annotation and Curation of Microbial Viruses, and Evaluation of Viral Community Function from Genomic Sequences.” *Microbiome* 8 (1): 90.
Kiełbasa, Szymon M., Raymond Wan, Kengo Sato, Paul Horton, and Martin C. Frith. 2011. “Adaptive Seeds Tame Genomic Sequence Comparison.” *Genome Research* 21 (3): 487–93.
Kurtz, Stefan, Adam Phillippy, Arthur L. Delcher, Michael Smoot, Martin Shumway, Corina Antonescu, and Steven L. Salzberg. 2004. “Versatile and Open Software for Comparing Large Genomes.” *Genome Biology* 5 (2): R12.
Letunic, Ivica, and Peer Bork. 2021. “Interactive Tree Of Life (iTOL) v5: An Online Tool for Phylogenetic Tree Display and Annotation.” *Nucleic Acids Research* 49 (W1): W293–96.
Li, Heng. 2021. “New Strategies to Improve minimap2 Alignment Accuracy.” *Bioinformatics* , October. https://doi.org/10.1093/bioinformatics/btab705.
Lowe, Todd M., and Patricia P. Chan. 2016. “tRNAscan-SE On-Line: Integrating Search and Context for Analysis of Transfer RNA Genes.” *Nucleic Acids Research* 44 (W1): W54–57.
Luo, Elaine, John M. Eppley, Anna E. Romano, Daniel R. Mende, and Edward F. DeLong. 2020. “Double-Stranded DNA Virioplankton Dynamics and Reproductive Strategies in the Oligotrophic Open Ocean Water Column.” *The ISME Journal*. https://doi.org/10.1038/s41396-020-0604-8.
“Maps: Draw Geographical Maps.” n.d. Accessed July 25, 2021. https://CRAN.R-project.org/package=maps.
Michniewski, Slawomir, Branko Rihtman, Ryan Cook, Michael A. Jones, William H. Wilson, David J. Scanlan, and Andrew Millard. 2021. “Identification of a New Family of ‘megaphages’ That Are Abundant in the Marine Environment.” *bioRxiv*. https://doi.org/10.1101/2021.07.26.453748.
Mistry, Jaina, Sara Chuguransky, Lowri Williams, Matloob Qureshi, Gustavo A. Salazar, Erik L. L. Sonnhammer, Silvio C. E. Tosatto, et al. 2020. “Pfam: The Protein Families Database in 2021.” *Nucleic Acids Research* 49 (D1): D412–19.
Moniruzzaman, Mohammad, Alaina R. Weinheimer, Carolina A. Martinez-Gutierrez, and Frank O. Aylward. 2020. “Widespread Endogenization of Giant Viruses Shapes Genomes of Green Algae.” *Nature* 588 (7836): 141–45.
Nayfach, Stephen, Antonio Pedro Camargo, Frederik Schulz, Emiley Eloe-Fadrosh, Simon Roux, and Nikos C. Kyrpides. 2021. “CheckV Assesses the Quality and Completeness of Metagenome-Assembled Viral Genomes.” *Nature Biotechnology* 39 (5): 578–85.
Nguyen, Lam-Tung, Heiko A. Schmidt, Arndt von Haeseler, and Bui Quang Minh. 2015. “IQ-TREE: A Fast and Effective Stochastic Algorithm for Estimating Maximum-Likelihood Phylogenies.” *Molecular Biology and Evolution* 32 (1): 268–74.
Ondov, B. D., Treangen, T. J., Melsted, P., Mallonee, A. B., Bergman, N. H., Koren, S., & Phillippy, A. M. 2016. Mash: fast genome and metagenome distance estimation using MinHash. *Genome biology* 17(1):1-14.
Paez-Espino, David, Emiley A. Eloe-Fadrosh, Georgios A. Pavlopoulos, Alex D. Thomas, Marcel Huntemann, Natalia Mikhailova, Edward Rubin, Natalia N. Ivanova, and Nikos C. Kyrpides. 2016. “Uncovering Earth’s Virome.” *Nature* 536 (7617): 425–30.
Parks, Donovan H., Maria Chuvochina, David W. Waite, Christian Rinke, Adam Skarshewski, Pierre-Alain Chaumeil, and Philip Hugenholtz. 2018. “A Standardized Bacterial Taxonomy Based on Genome Phylogeny Substantially Revises the Tree of Life.” *Nature Biotechnology* 36 (10): 996–1004.
Parks, Donovan H., Christian Rinke, Maria Chuvochina, Pierre-Alain Chaumeil, Ben J.

Woodcroft, Paul N. Evans, Philip Hugenholtz, and Gene W. Tyson. 2017. “Recovery of Nearly 8,000 Metagenome-Assembled Genomes Substantially Expands the Tree of Life.” *Nature Microbiology* 2 (11): 1533–42.

R Core Team. 2019. *R: A Language and Environment for Statistical Computing* (version 3.6.1). Vienna, Austria: R Foundation for Statistical Computing.

Reichardt, Jörg, and Stefan Bornholdt. 2006. “Statistical Mechanics of Community Detection.” *Physical Review. E, Statistical, Nonlinear, and Soft Matter Physics* 74 (1 Pt 2): 016110.

Roux, S., Emerson, J. B., Eloe-Fadrosh, E. A., & Sullivan, M. B. 2017. Benchmarking viromics: an in silico evaluation of metagenome-enabled estimates of viral community composition and diversity. *PeerJ* 5: e3817.

Shulgina, Yekaterina, and Sean R. Eddy. 2021. “A Computational Screen for Alternative Genetic Codes in over 250,000 Genomes.” *eLife* 10 (November). https://doi.org/10.7554/eLife.71402.

Sieradzki, Ella T., J. Cesar Ignacio-Espinoza, David M. Needham, Erin B. Fichot, and Jed A. Fuhrman. 2019. “Dynamic Marine Viral Infections and Major Contribution to Photosynthetic Processes Shown by Spatiotemporal Picoplankton Metatranscriptomes.” *Nature Communications* 10 (1): 1–9.

Sievers, Fabian, Andreas Wilm, David Dineen, Toby J. Gibson, Kevin Karplus, Weizhong Li, Rodrigo Lopez, et al. 2011. “Fast, Scalable Generation of High-Quality Protein Multiple Sequence Alignments Using Clustal Omega.” *Molecular Systems Biology* 7 (1): 539.

Sunagawa, Shinichi, Luis Pedro Coelho, Samuel Chaffron, Jens Roat Kultima, Karine Labadie, Guillem Salazar, Bardya Djahanschiri, et al. 2015. “Ocean Plankton. Structure and Function of the Global Ocean Microbiome.” *Science* 348 (6237): 1261359.

Wickham, Hadley. 2011a. “ggplot2.” *Wiley Interdisciplinary Reviews: Computational Statistics*. https://doi.org/10.1002/wics.147.

———. 2011b. “The Split-Apply-Combine Strategy for Data Analysis.” *Journal of Statistical Software* 40 (1): 1–29.

wwood. n.d. “GitHub - wwood/CoverM: Read Coverage Calculator for Metagenomics.” Accessed July 23, 2021. https://github.com/wwood/CoverM.
